# ‘Backpropagation and the brain’ realized in cortical error neuron microcircuits

**DOI:** 10.1371/journal.pcbi.1014164

**Published:** 2026-04-17

**Authors:** Kevin Max, Ismael Jaras, Arno Granier, Katharina A. Wilmes, Mihai A. Petrovici

**Affiliations:** 1 Neural Computation Unit, Okinawa Institute of Science and Technology, Onna, Japan; 2 Department of Physiology, Bern University, Bern, Switzerland; 3 Institute of Neuroinformatics, University of Zurich and ETH Zurich, Zurich, Switzerland; University of Surrey, UNITED KINGDOM OF GREAT BRITAIN AND NORTHERN IRELAND

## Abstract

Neural responses to mismatches between expected and actual stimuli have been widely reported across different species. How does the brain use such error signals for learning? While global error signals can be useful, their ability to learn complex computation at the scale observed in the brain is lacking. In comparison, more local, neuron-specific error signals enable superior performance, but their computation and propagation remain unclear. Motivated by the breakthrough of deep learning, this has inspired the ‘backpropagation and the brain’ hypothesis, i.e., that the brain implements a form of the error backpropagation algorithm. In this work, we introduce a biologically motivated, multi-area cortical microcircuit model, implementing error backpropagation under consideration of recent physiological evidence. We model populations of cortical pyramidal cells acting as representation and error neurons, with bio-plausible local and inter-area connectivity, guided by experimental observations of connectivity of the primate visual cortex. In our model, all information transfer is biologically motivated, inference and learning occur without phases, and network dynamics demonstrably approximate those of error backpropagation. We show the capabilities of our model on a wide range of benchmarks, and compare to other models, such as dendritic hierarchical predictive coding. In particular, our model addresses shortcomings of other theories in terms of scalability to many cortical areas. Finally, we make concrete predictions, which differentiate it from other theories, and which can be tested experimentally.

## Author summary

How does the cortex learn to perform complex functions? On the abstract level, this long-standing question in neuroscience has been addressed from multiple viewpoints. On the one hand, several studies have described how the error backpropagation algorithm, which revolutionized training of artificial neural networks, could be implemented on the “hardware” of the brain. It is however clear that the brain is not a simple feedforward neural network, but heavily relies on feedback connections, necessitating a more in-depth look at realistic cortical connectivity. On the other hand, classical predictive coding models propose that learning happens based on mismatches between expected and actual stimuli, and acknowledge the role of feedback and recurrent connectivity in the brain. However, such models also typically operate on a high level of abstraction, with unexplained information sharing between distant synapses and neurons, slow network dynamics, and phased learning. In this work, we introduce a biologically motivated, multi-area cortical circuit model, based on the connectome of the primate visual cortex. We show that our model can learn complex tasks using multiple cortical areas, while solving the aforementioned issues of bio-plausibility, and being demonstrably related to both the error backpropagation algorithm and the predictive coding framework.

## 1. Introduction

### 1.1. Background

The error backpropagation algorithm is the state of the art in training artificial neural networks (ANNs), with immense advances in practical applications made over the past decade. On the other hand, neuroscience has revealed great functional diversity across brain areas and an entire zoo of cell types. However, models taking into account such biological constraints have only produced limited understanding of how microscopic learning leads to complex function. Adapting individual components in a very complex network requires a meaningful local representation of errors; this has sparked the ‘backpropagation and the brain’ hypothesis [1,2], i.e., that biological neural networks learn via a form of the error backpropagation algorithm (BP) powering deep learning.

However, the biological implausibility of BP has been a long-standing point of objection [1,3]. Why would one then still consider backpropagation as a viable candidate for explaining learning in the brain? We argue that this is a consequence of applying Occam’s razor to physiological evidence: Mismatch responses (in other words: error encoding) have been observed in a variety of brain areas and across species, such as humans [4], non-human primates [5,6], mice [7–11], and birds [12]. In particular, experimental findings suggest that cortical layer 2/3 (L2/3) pyramidal cells (PYR) function as error coding neurons, with evidence across many cortical areas, e.g., visual areas [4,13–16], visuomotor [17], motor [18], auditory [19]. Error neurons have also shown to emerge naturally in simulated RNNs trained under constraints of energy consumption [20]. If cortex has explicit error units, and all neurons are plastic, then it should arguably use such errors to their fullest.

Currently, BP appears to be the most efficient way for training multi-area networks in terms of generalizability, scalability, and task complexity. This is because BP propagates local errors across all areas, tailored to each neuron according to its contribution to task performance (credit assignment). Since BP works so well on artificial networks, our aim is thus to discover whether the cortical hierarchy could implement a bio-plausible variant of the BP algorithm. In this work, we suggest a concrete circuit implementation, which provides experimentally testable predictions and performs well on many different benchmarks, and compare it to alternative suggestions.

### 1.2. Related work

A contentious point of discussion is the (in-)compatibility of BP with the predictive processing framework (predictive coding, PC). However, as highlighted by [21–25], there is a fundamental relationship between the computations performed in hierarchical predictive coding (hPC) and ANNs trained with error backpropagation. This relationship is well summarized by two properties. First, the structure of error propagation in hPC at equilibrium of neuronal dynamics is the same as the one of backpropagation, as first remarked by [21]. Second, the representations in hPC after neuronal inference and in BP after a forward pass are not necessarily the same, such that ‘vanilla’ hPC and BP do not lead to the exact same weight updates. This motivates a ‘fixed prediction assumption’, making the equivalence strict by using the activity induced by a forward pass in the learning rule of hPC. Multiple authors have also remarked that peculiar choices of hyperparameters (short inference with Euler-dt 1) also makes the equivalence strict [24,26]. Crucially, this means that the ‘backpropagation and the brain’ hypothesis and predictive coding provide similar solutions to the credit assignment problem. They can be seen as closely related learning algorithms, which have traditionally been employed on different task domains: predictive coding networks (PCNs) are commonly trained to predict stimuli, which is a generative task, while ANNs historically arose as solutions to classification problems. It is important to realize that the task domains are related by a simple exchange of stimuli and latent, and an inversion of the network hierarchy. Our model is built on this insight, and able to reflect it in its connectivity, with either a generative or classifier configuration (see Section 2).

While PCNs can be successfully trained on various tasks and scale to many areas [27,28], most studies do not detail how biology can implement the required computations. For example, the classical PC model by Rao and Ballard [29] is limited in its biological plausibility due to issues of ideal signal transport between neurons, weight copying, strictly hierarchical connectivity, and neuronal non-linearities (see Section 4.2).

Recent work aims to address these issues one by one. Two models stand out in particular due to their biological plausibility: the dendritic cortical microcircuit model of Sacramento et al. [30], and the dendritic hPC model by Mikulasch et al. [25], both of which propose that errors are constructed on dendrites of pyramidal cells via an interplay of local excitation and inhibition. This idea fits well into the theory of balanced networks and predictive coding, but assumes local interneurons *exactly* copying pyramidal neuron activity, which is unlikely given that interneurons integrate inputs from different cells, sometimes even non-linearly [31]. Moreover, models with dendritic error construction do not scale to more than two areas, as we will demonstrate using extensive simulations.

In contrast, we propose a model using recurrently connected prediction and error streams, loosely implementing the forward and backward pathways of error backpropagation. Similar connectivity is explored in attention-gated reinforcement learning (AGREL) and variants [32–36], but without solutions for the above-mentioned issues of bio-plausibility.

The most important computational achievement of our model is that it scales to many areas, while maintaining all of its bio-plausible constraints. Many studies in the literature have significantly simplified their model dynamics when scaling up simulations, e.g., by assuming ideal weight sharing [25,30], replacing neuron dynamics by steady-state approximations or artificially re-initializing voltages [30,37,38], or implicitly removing recurrency by calculating separate forward and backward passes [30,39]. Here, we will demonstrate that our model is able to scale without changing the implementation of its dynamics in any way.

### 1.3. Summary of our model

**Fig 1:**
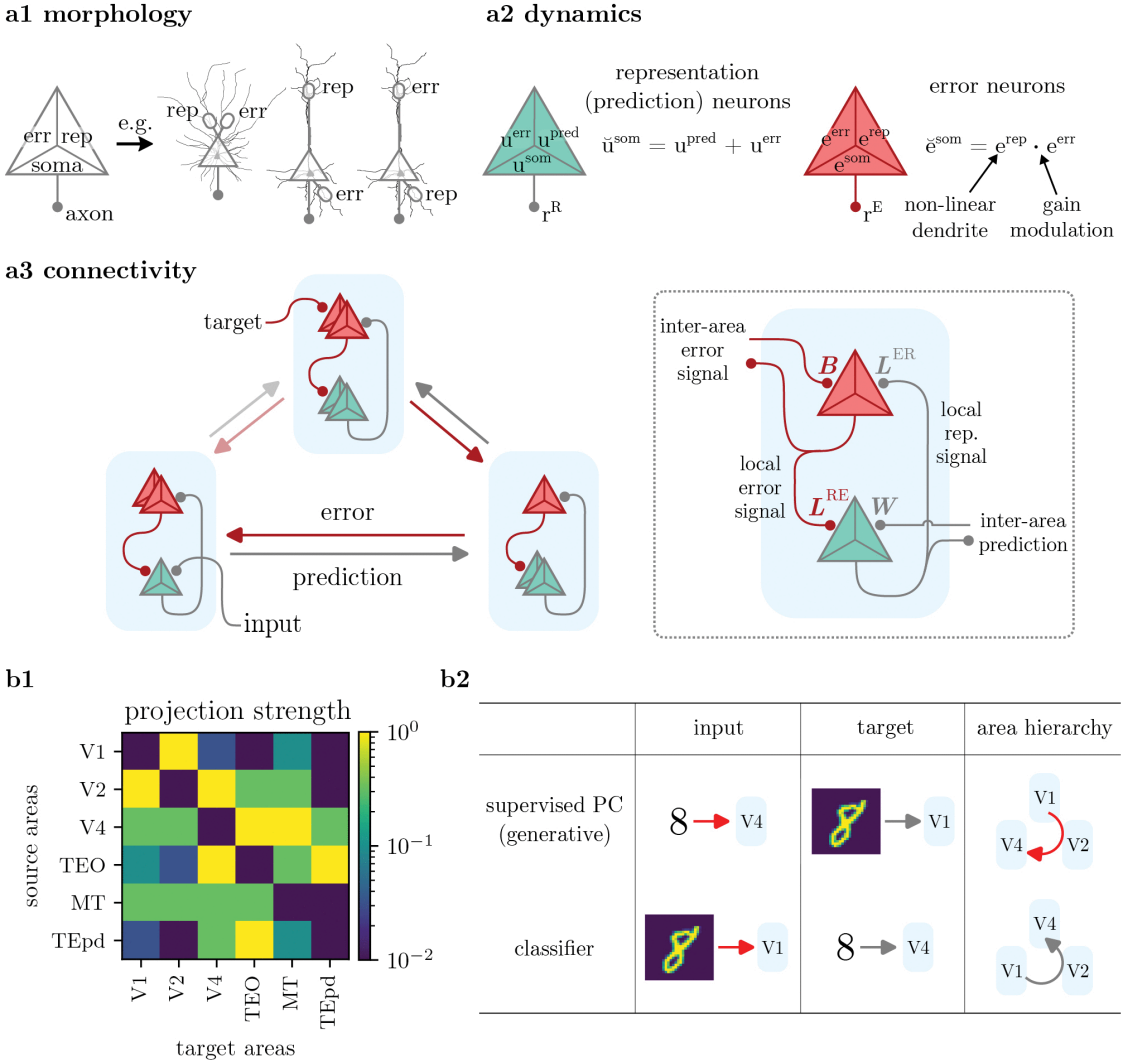
Neuron and network model. **a1)** Morphology: our model neurons have three compartments (soma, error and representation), which may be realized by various morphologies with apical, basal or perisomatic dendritic integration zones. **a2)** Dynamics: two types of pyramidal cells encode representations and errors, using distinct dynamics. **a3)** Connectivity: Between areas (left), two opposing streams of representation and error signals form, with preferential and weaker projections (opaque vs. semi-transparent arrows, respectively). Locally (right), representation units receive inputs from other areas onto their prediction-receiving compartment, and local error information on the error-receiving compartment. Error units integrate local representations and errors projected from other areas in their two dendritic compartments. **b1)** Projection densities across the Macaque visual cortex, used for the inter-area connectivity in our model. Reproduced from Markov et al. 2014 [40]. **b2)** Generative and classifier configurations of the model differ by inputs, targets, and area labels. In the (supervised) predictive coding configuration, the model receives a latent representation (‘input’) and learns to predict bottom-up stimuli (‘target’). *Errors* are projected downstream in the hierarchy of cortical areas. The ANN configuration has input and target exchanged, the task becoming digit classification. The information flow is inverted, and *representations* are projected downstream.

Our model addresses a large number of biological constraints, while establishing a clear relationship to BP. We model two distinct subpopulations of cortical pyramidal cells in each area: one error and one representation (= prediction) population, corresponding to L2/3 and L5 neurons respectively. Connections between error and representation units in different areas are based on the functional connectome of the primate visual cortex [40] (see Fig 1 b1). Thus, inter-area connectivity does not need to be strictly hierarchical, as opposed to most PC models in the literature. Furthermore, there is no biologically implausible one-to-one matching of prediction and error units within each area.

In order to integrate both predictions and errors, each neuron is modeled with three separate integration zones (‘compartments’): one somatic and two separate dendritic compartments. For example, neocortical pyramidal cells are characteristically defined by their somatic, basal and apical dendritic integration zones [41,42]. More generally, our model can be adapted to different neuronal morphologies, see Fig 1 a1. Neuron dynamics are implemented in continuous time, without discrete phases of information transfer (i.e., no distinct forward and backward passes as in ANNs and some bio-plausible models [30,32–35]). The dynamics of each neuron type, in conjunction with its connectivity, encode their function as either error or representation units. Error units make use of non-linear dendritic dynamics, and communicate locally within each cortical column, providing a neuron-specific learning signal to each representation unit. This is important, because it treats error units as distinct cells with their own dynamics, without requiring knowledge of the somatic potential of local representation units (see Section 4.2). All neurons implement prospective rate coding [43,44], modeling the diverse temporal signatures of neuronal outputs, and providing short-term memory to solve temporal tasks.

Learning is performed via a local Hebbian-type rule of the form presynaptic rate × postsynaptic voltage difference (delta rule). Similar to the voltage dynamics, learning is phaseless and always-on, as opposed to the vast majority of bio-plausible theories, such as PCNs [21–24,29], contrastive Hebbian learning [45,46], the wake-sleep algorithm [47], difference target propagation and variants [48–50], AGREL [33,51], and equilibrium propagation [37].

In this work, we will demonstrate the capabilities of our model to solve complex tasks and scale training to many cortical areas on par with ANNs, and compare in particular to the models of dendritic error construction [25,30]. Compared to existing bio-plausible theories of learning, the main novelty of this work is that it includes all of the following:

- successful learning without strict hierarchy of areas (→ *inter-area connectivity*);
- modeling of PYR as multi-compartment neurons with temporal dynamics (→ *morphology* ff.);
- phase-less, always-on learning (→ *plasticity*);
- no strict one-to-one matching of representation and error units (→ *plasticity*);
- efficient learning across many areas (→ *simulation results*).

## 2. Theory

We now introduce the architecture of our model, and how it describes learning in a network with multiple areas. Our model builds on the works of [30,43,44], inheriting the general architecture, neuron dynamics and synaptic plasticity rule of (Generalized) Latent Equilibrium, while adapting the bio-plausibility of the microcircuits by Sacramento et al. [30]. See Fig 1 for a summary of the architecture.

### 2.1. Inter-area connectivity

Like ANNs, PCNs derive computational efficiency from hierarchical implementations; however, biological networks are never strictly hierarchical. To accurately model the degree of hierarchy found in cortex, we base the inter-area connectivity of our network on the projection densities found among the first areas of the Macaque visual cortex [40]. There, one may describe a rough order between areas defined by the anatomical hierarchy of visual cortex [40,52,53] and the anterior temporal lobe [54]; however, functional connectivity shows projections also between non-neighboring areas (Fig 1 b1). For our model, this means that instead of a strict hierarchy, connections between all areas are allowed (the equivalent of skip connections in ANNs). This ‘loose hierarchy’ will allow us to compare learning performance to ANNs and strictly hierarchical PCN [25].

The network admits two configurations in terms of this ordering (Fig 1 b2): one where *errors* are passed from early to downstream areas (‘generative’ or ‘PC’ setup), and an inverted order, where *representations* are passed downstream (‘classifier’ setup). In the generative configuration, the task of representation neurons is to predict activity in primary areas, as in the Rao-Ballard model [29], albeit with different connectivity and neuronal dynamics (see Section 4.2). (We differentiate between the *principle* of predictive coding, i.e., a generative model where representations are projected top-down, and the specific predictive coding model by Rao and Ballard.)

In this section, we keep the discussion of our model general to the configuration, and refer to the streams as representation (green in Fig 1 a3) and error streams (red) (note that in the context of predictive coding, representation neurons are also known as *prediction* units). Our model is thus quite general and can encompass various hypotheses of cortical architecture, such as different directions of information flow or projection densities between and within areas.

Stimuli and latent activations are presented to the ‘input’ and ‘target’ areas, which are defined depending on the configuration (Fig 1 b2). For example, for the generative configuration, visual stimuli are presented to V1 as targets, while latent priors are provided to the downstream area V4 (‘input’); for sharp priors, e.g., in the form of labels, this amounts to supervised training. By modeling different latent priors, our model can also adapt other learning schemes, such as unsupervised learning as in the Rao-Ballard model (see Section 4.4).

### 2.2. Local neuron populations

In each area, we model two populations of pyramidal cells, acting as representation and error units (Fig 1 a2). These populations may be realized by cortical L2/3 PYR, corresponding to the error neuron populations (red in Fig 1), and L5 PYR, corresponding to the representation units (green). Across areas, information is passed between representation units through uni-directional synapses *W*, while errors are calculated at the ‘target’ area, and projected between error units through uni-directional weights *B* (Fig 1 a3, right). Within each area, representation and error units are connected via local weights *L*^ER^ (representation → error) and *L*^RE^ (error → representation), see Fig 1 a3.

We model each pyramidal neuron with three dendritic integration zones: a somatic, a representation and an error compartment. In real PYR, these compartments may be realized by a range of different morphologies, composed of, e.g., somatic, basal, perisomatic and apical integration zones. Depending on the type of neuron (representation or error unit), these compartments perform different calculations and observe different connectivity (Fig 1 a2), as we describe in the following.

### 2.3. Representation units: morphology

L5 PYR acting as representation units integrate all of their inputs in the soma, as in the classical neuron model of Petreanu et al. [55]. Importantly, to provide the correct error signal to a synapse *W* located at a representation-receiving dendrite, our model assumes that the somata *additively* integrate afferent error and representation signals (see *neuron dynamics*, below), with negligible non-linear distortions.

As illustrated in Fig 1 a1, such additive integration can be realized in biology by different morphologies. If both errors and representations are coded in perisomatic integration zones (Fig 1 a1, 1st morphology), the soma is able to sum the inputs with minimal non-linear effects.

However, when considering morphologies with long apical dendrites typical to L5 PYR (2nd & 3rd morphology), PSP dispersion and non-linear attenuation of somatic activity need to be taken into account. For example, if the representation neuron’s error-receiving compartment consists of a basal integration zone, and predictions are received in apical tufts (2nd morphology), the error signal provided to the synaptic site *W* will be attenuated after propagation along the apical dendrite. Conversely, error- and representation-receiving compartments may be swapped (3rd morphology), as hypothesized also in [30,56]. If error units are L2/3 PYR, this means that L5 PYR representation units receive local error signals through apical dendrites reaching L1, in line with experimental data [19,57]. However, in this morphology, apical errors are subject to dispersion when propagating to the soma. Furthermore, experiments have shown that top-down or local L2/3 input to L5 PYR non-linearly amplifies somatic activity [58,59], e.g., by triggering Ca^2+^-mediated plateau potentials.

In an alternative realization of our model, errors and representations are *both* received at the apical tuft. In this scenario, the additive integration could be performed by the apical tuft, and the sum would be propagated to the soma. This agrees with findings that the somata of L5 PYR are largely driven by apical tuft activity [42,58], and fits the results of Francioni et al. [56].

### 2.4. Representation units: neuron dynamics

Representation units integrate two streams of information: 1) representation units projecting from other areas onto a prediction-receiving compartment with voltage *u*^pred^; and 2) same-area error units projecting locally onto an error-receiving compartment with voltage *u*^err^ (see Fig 1 a3, inset). Each representation unit *additively* (Fig 1 a2, left) integrates both streams in its somatic voltage *u*^som^:


$$C_{m}\frac{\text{d}}{\text{dt}}u^{\text{som}}(t)=-g_{\text{l}}u^{\text{som}}-g^{\text{rep}}(u^{\text{som}}-u^{\text{pred}})-g^{\text{err}}(u^{\text{som}}-u^{\text{err}})\;,$$ (1)

where *C*_m_ is the capacitance of the soma, *g*_l_ is the leak of the soma, and {*g*^rep^, *g*^err^} are the conductances of the two dendritic compartments. Intuitively, the somatic voltage *u*^som^ is pulled towards *u*^pred^ with strength *g*^rep^, towards *u*^err^ with strength *g*^err^, and towards zero with strength *g*_l_. In other words, the relative size of these conductances determines how strongly the soma is affected by inputs from other prediction and error units, respectively. For $g^{\text{rep}}\gg g^{\text{err}}$, the soma of a representation neuron mostly encodes feedforward inputs from other prediction units, behaving like the units in an ANN. Thus, in the limit $\frac{g^{\text{rep}}}{g^{\text{err}}}\to 0$, our network dynamics can reproduce an ANN trained with BP; see Section 6.2 for an extended discussion. From these dynamics, one can further see that representation neurons have an effective membrane time constant $\tau_{m}^{r}\equiv \frac{C_{m}}{g_{\text{l}}+g^{\text{rep}}+g^{\text{err}}}$. For simplicity, we set the leak potential *E*_l_ to zero for all neurons in our model; this can easily be included by adding a term *g*_l_ *E*_l_ to all somatic dynamics.

### 2.5. Temporal dynamics

In our model, the neuronal output is an instantaneous firing rate *r*(*t*). Across nearly all instances in the literature, *r*(*t*) is modeled as a function φ of the current membrane potential *u*(*t*) only (e.g., [24,29,30,60]). However, this does not represent *t*he temporal expressiveness of real neurons well, whose responses also depend on the dynamics of stimulating currents [61–63]. This effect can, to some degree, be captured by the ‘prospective (!) coding’ mechanism [43,44,63], where firing rates are functions of *u*(*t*) and instantaneous rate of change $\frac{\text{d}}{\text{dt}}u(t)$. This means *t*hat the instantaneous firing rate is


$$r(t)=\varphi (\overset{˘}{u}(t))=\varphi (u(t)+\tau_{r}\frac{\text{d}}{\text{dt}}u(t))\;,$$ (2)

where $\overset{˘}{u}(t)$ is defined as the *prospective* voltage with time constant $\tau_{r}$. Various biological mechanisms can explain how neurons may compute $\overset{˘}{u}(t)$; e.g., coupling of adaptation currents as in the Izhikevich or AdEx models (see Section 6.3 in [44]).

**Fig 2:**
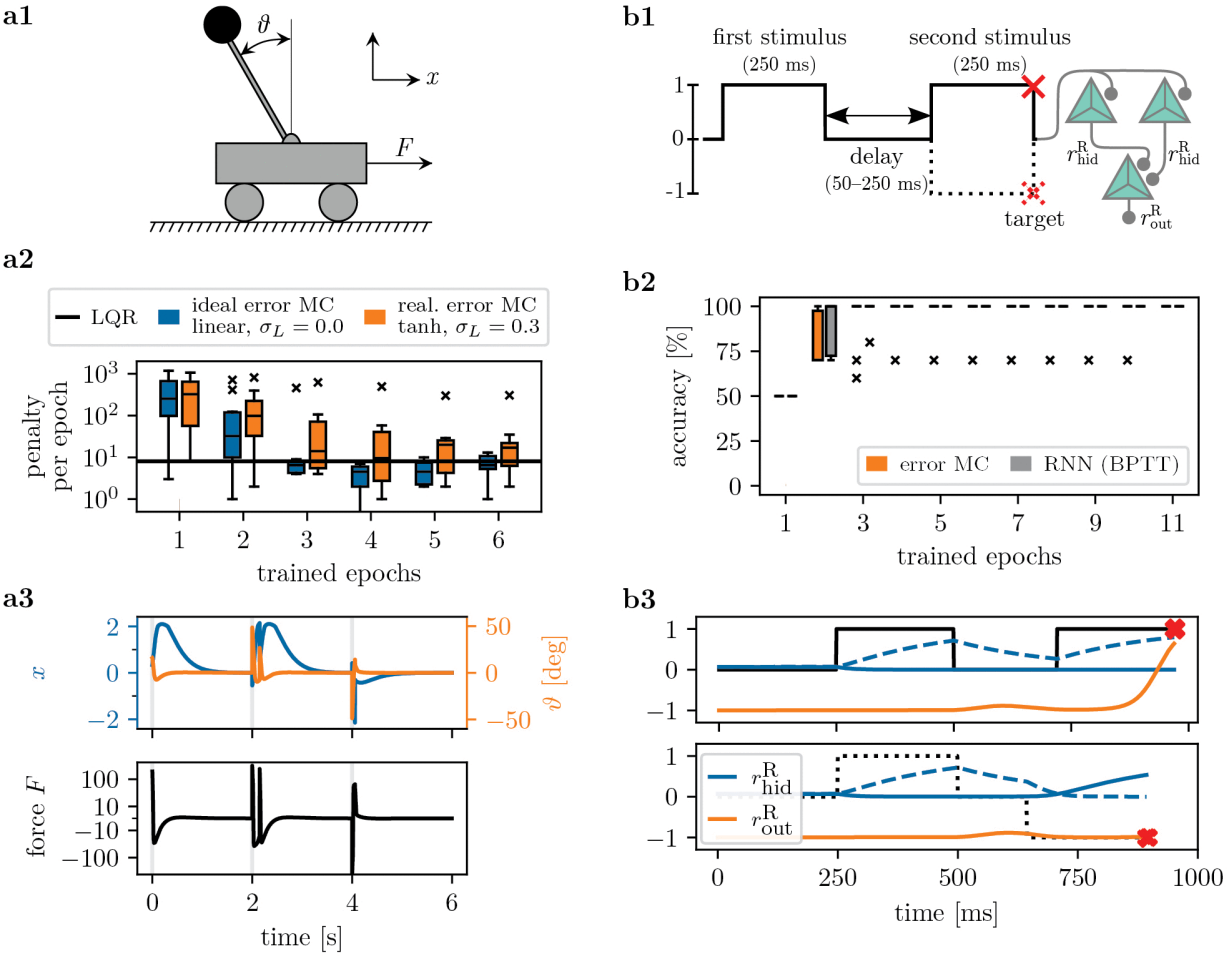
Error neuron microcircuits learn motor control and memory tasks. **a1)** Setup of cart pole task. The objective is to control the inverted pendulum such that ϑ→0 and x→0 are reached. **a2)** A microcircuit of size [4–1] successfully learns to stabilize the pendulum provided inputs $\left\{x,\dot{x},\vartheta ,\dot{\vartheta }\right\}$. We show the number of failed runs for the linear-quadratic regulator (LQR) and two error neuron microcircuits with different activation functions and local variability level $\sigma_{L}$ (one ‘ideal’ and one ‘realistic’ microcircuit setting). We show median and quartiles over 10 seeds. **a3)** Example trajectories for the ‘realistic’ microcircuit after training. The cart pole is reset every 2 s. **b1)** Delayed match-to-sample (DMS) task and network model. We show two of the four conditions with corresponding targets: ↑↑ (solid line) and ↑↓ (dotted). The time series is fed into a two-area network, each with [2–1] representation and error units. For both conditions, respective targets are marked by a red cross. **b2)** Accuracy during validation and testing for our model and an equivalent RNN trained with BPTT; median and quartiles over 10 seeds. **b3)** Example dynamics for ↑↑ and ↑↓ after training, showing the representation neuron rates $r_{\text{hid}}^{r}$ and $r_{\text{out}}^{r}$. Note the different length of the delay for each of the two conditions. The network makes use of the slow membrane $\tau_{m}^{r}$ as short-term memory.

The time constant $\tau_{r}$ determines the neuron’s temporal horizon for anticipating its own future firing rate: for fast information transfer, neurons may have approximately equal prospective and membrane time constants, $\tau_{r}\approx \tau_{m}$, such that the unavoidable lag introduced by the slow membrane is compensated by the prospectivity of the output. On the other hand, choosing smaller $\tau_{r}$ can implement neuron-wise short-term memory, enabling the network to solve temporal tasks (cf. DMS task, Fig 2). More generally, these time constants can be learned, enabling networks to adapt their memory to solve complex spatio-temporal tasks [43,44]. For simplicity, we restrict ourselves to fixed $\tau_{r}$ in this work. We denote the prospective firing rate of representation and error units as $r^{r}(t)=\varphi ({\overset{˘}{u}}^{\text{som}})$ and $r^{\text{E}}(t)=\varphi ({\overset{˘}{e}}^{\text{som}})$ respectively. Note that we use linear activations for error neurons in our model (see below).

### 2.6. Error units: morphology

Error units encode a neuron-specific error signal in their soma, which they provide to local representation units. In order to encode useful errors, they need to integrate backpropagated errors from other areas with information from local representation units, in the form of the derivative of the activation $\varphi^{\prime }({\overset{˘}{u}}^{\text{som}})=\frac{\partial \varphi ({\overset{˘}{u}}^{\text{som}})}{\partial {\overset{˘}{u}}^{\text{som}}}$. The integration of both signals is realized by one somatic and two dendritic compartments in each error neuron: one receiving errors from other areas, and a representation-receiving one for the reconstruction of $\varphi^{\prime }$. Assuming that error units are L2/3 PYR, their morphology may also follow the examples in Fig 1 a1.

However, the error backpropagation algorithm requires that the soma integrates the *product* of the two dendritic signals – as opposed to their *sum*, as in L5 representation units. Error units could use gain modulation to implement such multiplicative dynamics, similar to the mechanism observed in L5 PYR [58,59,64]. Note that it is also possible that error neurons only have a single dendritic compartment, which would be gain modulated by $\varphi^{\prime }$, without explicit encoding of the rate derivative in a dendritic compartment.

### 2.7. Error units: neuron dynamics

The soma of an error unit *e*^som^ *multiplicatively* integrates its dendritic compartments (Fig 1 a2, right),


$$C_{m}\frac{\text{d}}{\text{dt}}e^{\text{som}}=-g_{\text{l}}e^{\text{som}}-g^{\text{den}}(e^{\text{som}}-e^{\text{rep}}\cdot e^{\text{err}})\;,$$ (3)

where *g*^den^ denotes the effective conductance coupling of both compartments to the soma, *e*^rep^ is the voltage of the compartment receiving local representations, while *e*^err^ is the compartment receiving error signals from other areas (cf. Fig 1 a3, inset). Intuitively, the somatic voltage *e*^som^ is pulled towards $e^{\text{rep}}\cdot e^{\text{err}}$ with strength *g*^den^, and towards zero with strength *g*_l_. The modulation factor $\varphi^{\prime }({\overset{˘}{u}}^{\text{som}})$ can be reconstructed in the dendrite using temporal noise ξ(t) propa*g*ating in the network, and the use of non-linear dendrites (see Methods, Section 6.4). For simplicity, we summarize the effective computation performed in both compartments as $e^{\text{rep}}\cdot e^{\text{err}}=[L^{\text{ER}}\;\varphi^{\prime }({\overset{˘}{u}}^{\text{som}})]\cdot \sum_{\mathrm{pre}}B\;r_{\mathrm{pre}}^{\text{E}}$.

Note that in our model, error signals are explicitly *projected* across areas, implementing the BP step $\delta_{\ell }=\varphi^{\prime }(u_{\ell })⊙[W_{\ell +1,\ell }{]}^{T}\;\delta_{\ell +1}$ (see Equation 6). This differentiates our model from other PCNs [21–25,29], where errors are computed within each area as a local *difference* between top-down prediction and bottom-up stimulus. As we will demonstrate, this has benefits in terms of performance (Section 3.2) and biological plausibility (Section 4.2).

In this work, we do not address the handling of signed error signals. As neuronal outputs can only be non-negative, we need to explain how an error unit can communicate both positive and negative errors. This may be addressed by distinct populations for positive and negative errors [65,66], or transformed learning signals [66–70], see Section 4.4. For simplicity, we here assume linear activation functions for all error units, i.e., $\varphi ({\overset{˘}{e}}^{\text{som}})={\overset{˘}{e}}^{\text{som}}$.

### 2.8. Synaptic plasticity

We move on to the theory and learning rules for the weights, that is: *W* projecting representations, *B* projecting errors, and local weights *L*^RE^ and *L*^ER^.

Representation-projecting weights *W* are the functionally relevant connections in the network, i.e., these weights are trained to minimize the cost of a given task. To model the cortical connectome, we scale connectivity between different pairs of areas in *W* using the projection density of the Macaque visual cortex, see Fig 1 b1. For learning of *W*, we use a form of the delta rule:


$$\frac{\text{d}}{\text{dt}}W=[{\overset{˘}{u}}^{\text{som}}-\frac{g^{\text{rep}}}{g^{\text{tot}}}u^{\text{pred}}]\;r_{\mathrm{pre}}^{\text{T}}\;,$$ (4)

where $\frac{g^{\text{rep}}}{g^{\text{tot}}}$ is a conductance-weighting factor, reflecting the coupling between soma and dendrite (see Methods). This is a local error-correcting learning rule based on the product of a postsynaptic error ($\left[{\overset{˘}{u}}^{\text{som}}-\frac{g^{\text{rep}}}{g^{\text{tot}}}u^{\text{pred}}\right]$) and the presynaptic rate ($r_{\mathrm{pre}}^{\text{T}}$). Similarly to the work by [71], this can be interpreted as learning with the dendritic prediction of somatic voltage. Note that the postsynaptic term encodes exactly the error signal required by the backpropagation algorithm at the synapse. In particular, for quasi-instantaneous neurons ($\tau_{m}^{r}=\tau_{r}^{r}$) in a hierarchical setup with ideal local weights $L^{\text{ER}}=L^{\text{RE}}=1$, we have


$$[{\overset{˘}{u}}^{\text{som}}-\frac{g^{\text{rep}}}{g^{\text{tot}}}u^{\text{pred}}]\propto \varphi^{\prime }({\overset{˘}{u}}^{\text{som}})⊙B{\overset{˘}{e}}_{\text{post}}^{\text{som}}\;,$$ (5)

recovering the exact structure of error backpropagation (see Methods, equations 6 and 7, and equation 17 for more details on the derivation). For different time constants ($\tau_{m}^{r}>\tau_{r}^{r}$), error neurons are able to compensate the lag of their partnering representation units, as described in [44].

Local weights are static in our model, and we assume that representation and error units are coupled by preferential targeting – i.e., there is only a loose one-to-one matching between both populations in each area. To model this, we initialize *L*^RE^ and *L*^ER^ as identity matrices with uniform noise, $L^{\text{RE}}=1+𝒰(-\sigma_{L},\sigma_{L})$ and equally for *L*^ER^. In Section 3, we demonstrate how the network benefits from such loose matching.

Error-projecting weights: Exact backpropagation, like many realizations of predictive coding [21–23,25], requires symmetric weights for the transport of representations and errors across areas, i.e., $B_{\ell ,m}=[W_{m,\ell }{]}^{T}$. This is known as the *weight transport* problem, where two distant areas are required to share their synaptic weight information. Many solutions have been proposed in the literature, such as the Kolen-Pollack algorithm [72], Feedback Alignment [73] or Phaseless Alignment Learning (PAL) [74]. The latter harnesses intrinsic neuronal noise ξ(t) to train error-transporting weights $B_{\ell ,m}$ to approximate $[W_{m,\ell }{]}^{T}$, similarly to how we reconstruct $\varphi^{\prime }({\overset{˘}{u}}^{\text{som}})$ in the error units. Furthermore, PAL is fully compatible with cortical microcircuit models, as demonstrated in [74]. Due to these two reasons, PAL is particularly suited to solve the weight transport problem in our model. It can be implemented by endowing the error-receiving synapses with the learning rule $\dot{B}(t)=\xi (t)\;{\hat{r}}_{\mathrm{pre}}^{\text{E}}(t)-\alpha \;B$, where ξ(t) is the noise on the potential of the post-synaptic error unit, ${\hat{r}}_{\mathrm{pre}}^{\text{E}}(t)$ is the high-pass filtered, pre-synaptic rate of bottom-up error units, and α a scalar factor controlling homeostatic regularization. Note however that for computational feasibility, we here approximate PAL by setting error-projecting weights to a noisy transpose of representation weights (see Methods).

Finally, we stress that all information is processed simultaneously in our network (no phases), all information is locally available at the synapse, and learning is always-on.

### 2.9. Differences to classical PCN

Due to the equivalence of predictive coding and backpropagation under the ‘fixed prediction’ assumption (see Section 4.3), our model is also an implementation of predictive coding. In particular, for ideal local weights, it is computationally equivalent to classical PC models [29,69], but differs in its inter-area connectivity and neuronal dynamics. Most apparent is that we have two separate prediction and error streams, while classical PCNs have bidirectional projections between error and representation units (see also Fig 7). Why do we implement such connectivity? Our model has a number of advantages:

(i) Faster and less noisy: predictions don’t need to go through error units in our model, which would double the number of neurons involved in an inference step – this affects processing speed and adds noise sources.
(ii) Simple switching between inference and learning: as the error and prediction streams are only loosely coupled, error signals can be introduced dynamically, without the need to wait for the dynamics of all neurons to re-settle. This may be particularly important for tasks where learning signals are only provided sparsely, such as reinforcement learning.
(iii) Signal transport and local weights: in most rate-based PCNs, error units compute the local error as a difference of voltages, requiring (bio-implausible) access to the somatic *voltage* of representation neurons. Alternatively, errors can be defined on the level of firing rates, requiring then that error units have access to the exact activiation function of representation units. In either case, following the exact derivation of PC dynamics based on gradient descent of an optimization function, it appears that error units serve as linear, one-to-one pseudo-compartments of representation units instead of actual neurons.

In our model, errors are explicitly projected from area to area by the error neurons, without the need to compute differences in every error neuron. These errors are then projected locally to representation units, which make them available at the synaptic site *W*.

## 3. Results

### 3.1. Motor control and spatio-temporal credit assignment

To demonstrate the capabilities of the error neuron microcircuit model for functional learning, we first train our implementation on two small benchmarks. The first is the cart pole task (Fig 2 a), a classical motor control problem also known as the inverted pendulum. The network receives four inputs: the current position and velocity of the cart ($x,\dot{x}$), and angle and angular velocity of the pole ($\vartheta ,\dot{\vartheta }$). The aim is to produce a motor output (force *F*) which achieves an upright position of the pole (ϑ=0) and navigates the cart to the position *x* = 0, as illustrated in Fig 2 a1. The cart pole is initialized with x~𝒰(−1,1) and $\theta \sim 𝒰(-57^{\circ },+57^{\circ })$.

We train the networks to reproduce the motor control of a linear-quadratic regulator (LQR), an algorithm that optimizes control for linear systems, and test performance using a simulation of the non-linear dynamics of the cart pole. Performance is measured by the penalty log, i.e., how often the pole falls or fails to reach the position *x* = 0. As Fig 2 a2 shows, error neuron microcircuits with five representation and error units each learn to solve the task, on par with the LQR in terms of the penalty log. We demonstrate this both with the ‘ideal’ microcircuit, which has linear neuronal activation and perfect one-to-one matching between error and representation units, as well as the more realistic model, with non-linear activations $\varphi ({\overset{˘}{u}}^{\text{som}})$ of representation units, and variability $\sigma_{L}$ on local weights. See Fig 2 a3 for example trajectories of a trained error neuron circuit.

Next, we show that error neuron microcircuits can solve tasks requiring memory (Fig 2b). We use the delayed match-to-sample (DMS) task [75], equivalent to temporal XNOR. The task is to compare two sequential stimuli to identify if the stimuli match sign (↑↑ or ↓↓) or have opposing signs (↑↓ or ↓↑), see Fig 2 b1. The input is presented to the network as a one-dimensional time series, and therefore requires short-term memory to be solved. Task complexity is increased by an intermittent delay period with variable length.

In our model, short-term memory is encoded by the leaky integration of the membrane of representation units, and tunable memory length is achieved by the *prospective* coding mechanism (see Section 2). Task-specific neuronal time constants can be learned, as demonstrated in [43,44]; however, we here only assume a loose agreement between the timescale of the task and neuronal time constants, and thus set $\tau_{m}^{r}\approx 220$ ms; such a large time constant can be provided by neuronal adaptation [44,63]. Fig 2 b2 shows that our model can solve the task using only six neurons (three representation, three error units); example traces are shown in Fig 2 b3. For comparison, we also train an equivalent layered RNN (two hidden units and one output neuron with self-recurrent connections, in lieu of the leaky integration of our representation neurons). We observe that our circuit performs on par with the RNN trained with backpropagation-through-time (BPTT).

### 3.2. Explicit error neurons scale to many areas, where dendritic error construction struggles

Our model differs from the bio-plausible models of error learning by Sacramento et al. [30] and dendritic hierarchical predictive coding (dendritic hPC, Mikulasch et al. [25]) through the existence of explicit error neurons, as in classical predictive coding models [29] (albeit without local difference calculation at every area). Here, we compare these models to determine advantages of explicit error neurons.

**Fig 3:**
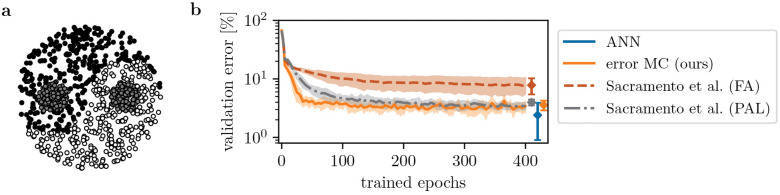
Error neuron microcircuits learn to solve a non-linear classification task. **a)** Visualization of the Yin-Yang dataset, with each x-y position corresponding to one of three classes (yin, yang, dot). **b)** Validation and final test error of the error microcircuit model with network size [30–3]. We compare to the model by Sacramento et al., implemented with feedback alignment (FA) and approximate weight transport like in our model (PAL); these results are reproduced from [74]. We also show the test error in an ANN trained with BP with equal network size. The shading indicates mean and standard deviation over 10 seeds.

In Fig 3, we show performance of our model on the Yin-Yang task [76], a non-linear classification task with three classes (visualized in Fig 3a). We compare performance to an ANN trained with BP, and the dendritic microcircuit model of Sacramento et al. [30], in its vanilla form (Sacramento et al. (FA)) and with approximate weight transport (Sacramento et al. (PAL)), the latter of which is analogous to the implementation in our model. As Fig 3 b show, our model performs on par with the dendritic microcircuit model with PAL, and nearly reaches the accuracy of the ANN.

**Fig 4:**
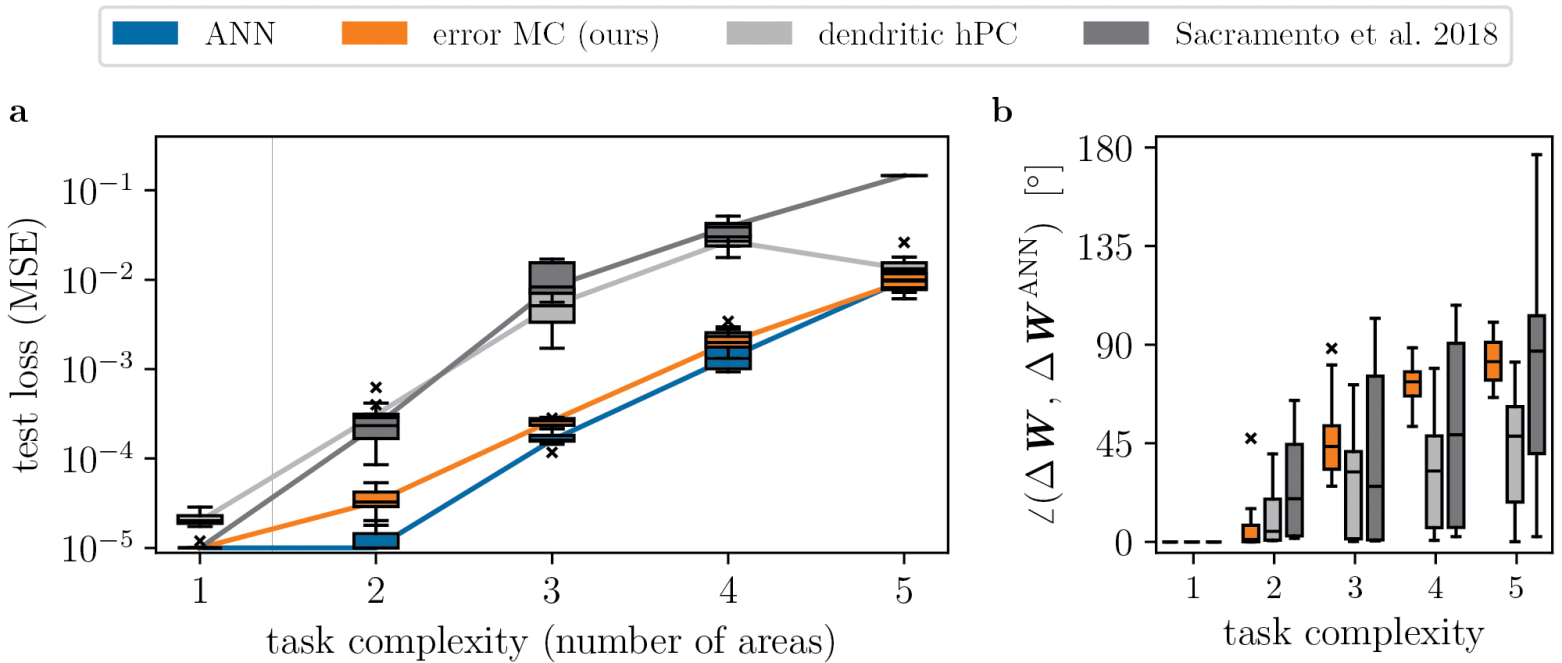
Error neuron microcircuit trains many areas, on par with an ANN. We compare learning in our model to an ANN trained with backpropagation, and the dendritic error computation models of Mikulasch et al. [25] and Sacramento et al. [30]. In order to show that our model scales to large networks, we systematically increase the number of areas. The task is to imitate a teacher network of the same architecture, where task complexity increases with number of areas. **a)** Mean loss on test set (median and quartiles over 10 seeds; performance before training is $𝒪(10^{-1})$ across models and task complexities). Our error neuron microcircuit performs on par with the ANN, while the dendritic hierarchical PC model of Mikulasch et al. [25] and the microcircuit model of Sacramento et al. [30] perform more poorly when training more complex tasks. **b)** Alignment between synaptic weight changes in all three models vs. those of the ANN (median over all seeds and areas).

In Fig 4, we demonstrate that our model scales to training of many areas, and performs on par with an ANN of equivalent size. The networks are trained to learn an input-output mapping recorded from a randomly initialized teacher ANN with the same architecture; therefore, task complexity increases with the number of areas in the networks. This guarantees that the student networks are in principle able to solve the task, but also that good performance requires training of all areas – in particular, it is not sufficient to train only the output weights.

We again compare performance to the model of Sacramento et al. with approximate weight transport, and also include dendritic hPC [25] in these experiments. To ensure a fair comparison, we have generalized dendritic hPC to a non-linear version in this work (see Section S1.2 in S1 Text); however, as we find that the non-linear version performs similarly to the linear model (Fig D in S1 Text), we have used the original model of [25] here.

We find that the two models with dendritic error construction do not scale well to multi-area tasks, indicating that dendritic error construction fails when errors are projected across more than two areas (Fig 4 a). On the other hand, our networks with explicit error representations always perform on par with the baseline, ANNs trained with backpropagation. This is an important result, indicating that current bio-plausible models of dendritic error computation lack the scalability required to explain learning across the cortical hierarchy (see Discussion).

Note that task complexity 5 cannot be fully solved by any network, as even the ANN baseline is only able to reduce the loss by about one order of magnitude. Indeed, training only the last three areas of the ANN achieves about the same performance, with a test loss of $(1.2\pm 0.1)\cdot 10^{-2}$. This indicates that the networks are not able to make use of learning in all of their five areas, which explains why all models perform equally on this task (barring the model of Sacramento et al.).

Further, our model differs from the others by incorporating connections between all areas. To ensure that this is not the reason of its higher performance, we have repeated the experiment with strictly hierarchical connectivity only, see Fig A in S1 Text. Indeed, the networks with strictly hierarchical connectivity perform equally well.

In Fig 4 b, we show the alignment between weight dynamics of the different models compared to the layered ANN. In the models of [25] and [30], larger deviations from BP are correlated with decreased performance. Such deviations can be explained, e.g., by 1) violation of the ‘fixed prediction’ assumption (see Section 4.3), and 2) imperfect lateral inhibition, generating ‘wrong’ error signals in the dendrites. At least for the model of Mikulasch et al., we have identified the latter to be the cause of the large alignment angles and drop in performance, cf. Fig B in S1 Text.

Curiously, in our model, we do not observe a correlation between performance and weight trajectories compared to BP. This applies even for the strictly hierarchical implementation (Fig A b in S1 Text), hinting that neural dynamics do not need to obey the ‘fixed prediction’ assumption for successful learning (cf. Section 4.3).

### 3.3. Networks benefit from loose matching of error and representation units

An essential feature of our model are the populations of error and representation neurons in each area. Here, the precise one-to-one matching of prediction and error neurons usually found in PCNs is replaced by preferential targeting, where local weights are not restricted to an identity matrix. As we will demonstrate, this does not only improve biological plausibility, but also impacts task performance.

**Fig 5:**
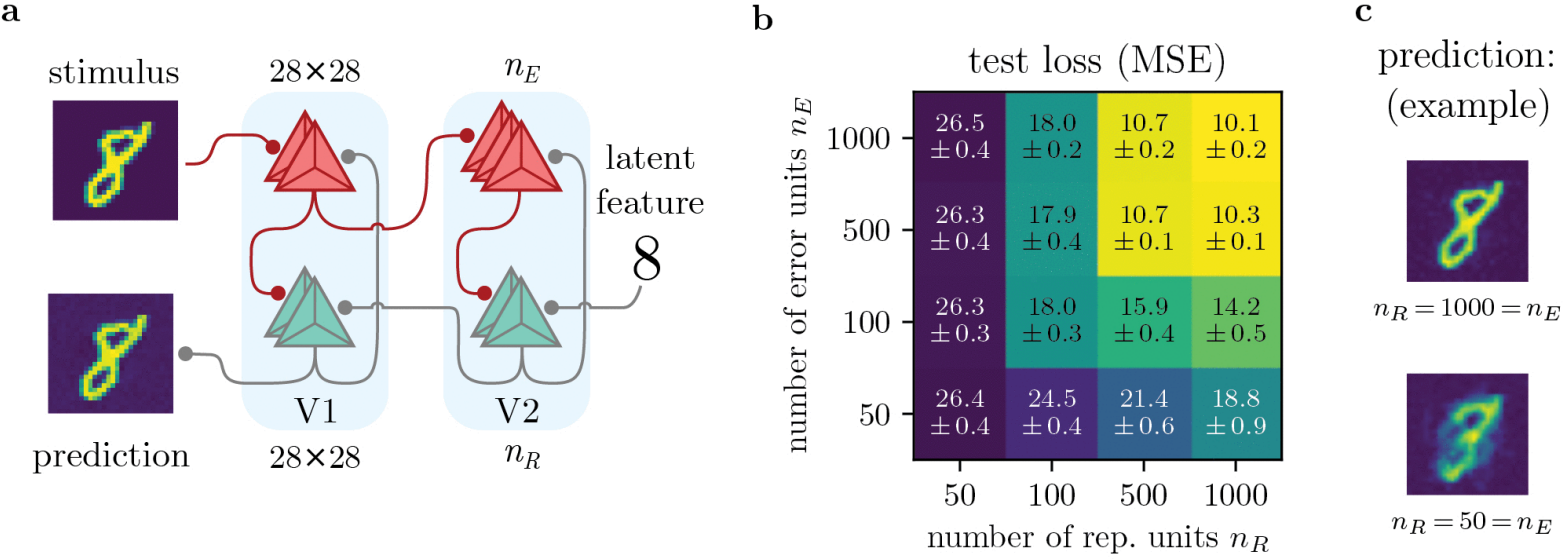
Implementation of predictive coding, and stability under variation of neuron populations. We demonstrate that our model accommodates predictive coding using a generative task, where the top-down input (‘latent feature’) is a digit [0,...,9], and the desired prediction a 28 × 28 image from the MNIST dataset. **a)** Network setup. As opposed to previous tasks, the network is inverted, thereby implementing stimulus prediction. V1 is modeled with 784 representation and error neurons each, whereas in V2, the number of representation units *n*_*R*_ and error units *n*_*E*_ can be varied. **b)** Test loss for different *n*_*R*_, *n*_*E*_ (mean and stdev over 10 seeds). Performance generally increases with larger populations, but is stable even for $n_{R}\neq n_{E}$. Note that the networks benefit from additional representation units (*n*_*R*_ > *n*_*E*_, lower right). **c)** Example predictions of the digit ‘8’.

We train a two-area network representing the early visual areas (e.g., V1 and V2), with 784 (28 × 28) representation and error neurons each in V1, and a variable number of *n*_*R*_ and *n*_*E*_ neurons in V2; see Fig 5a. Representation neurons are tasked to predict activity in V1 matching the label provided as fixed top-down input. This deterministic, generative network is therefore an implementation of supervised predictive coding. The images fed into the network are 28 × 28 images of the MNIST dataset (one image per digit class), while top-down latent features are provided through one-hot encoding of the corresponding classes.

As Fig 5 b & c show, the networks successfully learn to predict visual stimuli given a latent feature. To make sure that the network is actually required to propagate errors, we disabled learning of *W* from the top-down latent feature to V2, and indeed find drastically reduced performance with a test loss of 40.3 ± 0.4 (*n*_*R*_ = 1000 = *n*_*E*_). Finally, an ANN trained on the task achieves similar performance to our model, for which the test loss is 9.2 ± 0.1 (*n*_*R*_ = 1000).

As one expects, increasing the number of representation and error units in our model leads to better predictions (diagonal in Fig 5 b). Interestingly however, the networks’ performance is also stable under variation of population numbers. For example, for all combinations of $n_{R}=\{500,1000\}$ and $n_{E}=\{500,1000\}$, the mean square error (MSE) is approximately the same.

Notably, additional representation units are more useful than extra error units: For a given number of error units, increasing the pool of representation units *always* improves performance, whereas more error neurons are only beneficial if there already is a sufficient number of representation units (*n*_*R*_ ≥ *n*_*E*_).

Why does relaxing of strict one-to-one matching not deteriorate performance, but in fact improve it for *n*_*R*_ > *n*_*E*_? Of the *n*_*R*_ representation units, only *n*_*E*_ effectively receive an error signal, and thus learn; but while the remaining (*n*_*E*_−*n*_*R*_) representation units do not learn, they still increase the dimensionality of the represented data. Similar to a high dimensional feature space in a support vector machine, such higher dimensional representations can be exploited by downstream areas for improved learning (see S1.7 in S1 Text for details).

These results imply an experimental prediction, where a larger pool of representation units is generally favored over a larger number of error units (see Discussion).

### 3.4. Error neuron microcircuits are robust to neuron variability and ablations, unless error signals become too noisy

**Fig 6:**
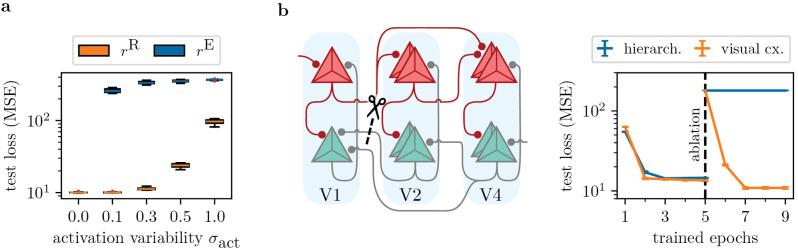
Effects of neuron variability and ablations under the generative MNIST task. **a)** We model neuron variability by adding parameter variability to the offset and slope of each neuron’s activation function. We sample from a normal distribution with standard deviation $\sigma_{\text{act}}$ and mean 1 and 0 for slopes and offsets, respectively. Adding variability only to representation units (orange) shows their resilience even to large variability (e.g., $\sigma_{\text{act}}=0.3$). However, as the networks rely on correct errors to be transported, they display high sensitivity to additional variability on error neuron activations (blue). **b)** We extend the setting of Fig 5 to three cortical areas (V1, V2 and V4), and train two implementations of our model: one using strictly hierarchical connectivity, and one using the visual cortex connectivity with skip forward and backward connections (Fig 1 b1). After initial training, we ablate connectivity from V2 to V1, and disable learning of new weights between these areas. While the hierarchical model is now unable to solve the task, the visual cortex model makes use of the skip connections to recover its performance. All points show mean and standard deviation over 10 seeds.

To assess the impact of parameter variability and ablations on our model, we again consider the generative MNIST task of Fig 5. First, we model variability of neuron activations φ in a network with [784–1000] representation units, and the same number of error neurons (Fig 6 a; note that error units still are linear, $\varphi ({\overset{˘}{e}}^{\text{som}})={\overset{˘}{e}}^{\text{som}}$). We introduce neuron-wise variability to the slope and offset (bias): activation slopes are noised by sampling from $𝒩(1,\sigma_{\text{act}})$, where the standard slope used in the other experiments corresponds to 1. Similarly, offsets are noised using $𝒩(0,\sigma_{\text{act}})$.

Fig 6 a shows that network performance is rather stable under variability of representation neurons, $r^{r}=\varphi ({\overset{˘}{u}}^{\text{som}})$. This can be explained by the algorithmic power of the error backpropagation algorithm: as long as errors are calculated correctly (no variability on $r^{\text{E}}=\varphi ({\overset{˘}{e}}^{\text{som}})$), representation unit weights are trained to solve the task, independent of their activation function. Only for very large variability, the representation units are unable to learn the task in general, e.g., due to vanishing gradients caused by large slope factors (for which $\varphi^{\prime }({\overset{˘}{u}}^{\text{som}})\approx 0$ for most somatic voltages).

Variability on the error units on the other hand represents a fundamental challenge: In that case, representation units may possess the capabilities of solving the task, but are not guided to a solution due to faulty error signals. We see this reflected in the failure to train already at small levels of variability on $r^{\text{E}}=\varphi ({\overset{˘}{e}}^{\text{som}})$. In Fig C in S1 Text, we demonstrate that this can be fully attributed to offset variability – the results can be interpreted using the framework of sign-concordant feedback alignment [77], for which we refer to the Discussion.

Finally, we study how ablations affect our microcircuit model (Fig 6 b). In strictly hierarchical models of cortex, the disruption of synaptic weights between two areas fatally hinders prediction and learning. Due to our inclusion of realistic connectivity based on visual cortex, other areas may learn to take on the role of the ablated connectivity. We extend our model implementing the generative MNIST task to three areas, modeling V1, V2 and V4 with [784-500-500] representation neurons, and an equal number of error units. After initial training using the full connectome, we ablate the weights *W* projecting representations from V2 to V1, and disallow growth of new synapses between these areas. Upon continuing training, we see that the synapses projecting from V4 to V1 are able to take on the role of the ablated connections, and fully recover predictive performance. As expected, a strictly hierarchical implementation fails to recover, as there is no way for V2 or V4 to project to V1 anymore.

## 4. Discussion

### 4.1. Experimental evidence and predictions

By integrating our novel mechanisms with aspects from previous works, we are able to address a wide range of bio-plausibility issues of the ‘backpropagation and the brain’ hypothesis; for a comprehensive list, see Table 1. In the following, we will describe concrete measurable correlates which will be able to differentiate our hypothesis from other models of learning in cortex.

**Table 1:** Translation of error backpropagation to bio-plausible model. We describe issues of bio-plausibility in ANNs and the BP algorithm (first section), and general issues of models of learning in cortex (second section). The last entries show important issues not addressed in this work, and potential solutions.

bio-plausibility problem of BP	our solution
BP has separate inference and learning phases; buffering of inference activations is required for learning phase	our model is fully dynamical with always-on learning
ANNs operate under ‘fixed prediction’ assumption, while in-vivo neural activity is strongly influenced by learning signals	we introduce tunable nudging strength *g*^err^, allowing for BP-like and target-based learning (small vs. large *g*^err^)
**general bio-plausibility issue**	**our solution**
artificial neurons are point-like without internal dynamics	multi-compartment neurons with leaky integration and prospective coding, modeling a wide range of realistic neuron dynamics [63]
explanation gap between predictive processing and error backpropagation in terms of applicable tasks (stimulus prediction, minimization of arbitrary loss) and architecture	mathematical equivalence of PC and BP [21–24], while architecturally, our model allows two configurations: generative and classifier setup (Fig 1)
error-projecting weights need to be transposes of representation weights $W_{\ell ,\ell -1}$ (weight transport problem)	we approximate PAL [74], which learns error projections using temporal noise and a local learning rule
many PCNs implement only strictly hierarchical connectivity	our model features realistic inter-area connectivity based on visual cortex
leaky-integrator dynamics can lead to slow inference and bad error signals before steady state is reached (relaxation problem [43])	prospective coding of rates, $r=\varphi (\overset{˘}{u})$
where are errors encoded?	explicit error neurons *r*^E^
many PCN implement biologically implausible one-to-one matching of errors and representations	two separate populations with free numbers of neurons
how are errors provided to representation units?	realistic local connectivity
within representation units, how does the error reach the correct synapse?	delta rule (alternatively: Urbanczik-Senn learning rule [71])
how is $\varphi^{\prime }(u_{\ell })$ calculated?	we introduce a method for local construction of $\varphi^{\prime }$, using the same principle as PAL
**general bio-plausibility issue**	**potential solution**
*rate-based model*	*spiking microcircuit implementation, e.g., spiking sampling neurons as in [78]*
*temporal tasks: knowledge of future dynamics (BPTT) or large memory requirement (RTRL)* [79]	*prospective error coding (Generalized Latent Equilibrium* [44])
*general, recurrent connectivity instead of layered architecture with skip connections*	*bio-plausible approximation to BPTT [44], allowing for learning in general, recurrent microcircuit architectures*
*Dale’s law*	*separate E-I units within representation and error populations*
*signed error signals*	*E-I units with local inhibition, transformed errors, or relative to bias [65–69,80,81]*
*dendritic nonlinearities, PSP dispersion during propagation, BAC firing*	*more bio-realistically detailed neuron model with functional explanation for complex dendrite dynamics*

Generally, our model allows for two possible configurations: the (generative) predictive coding implementation, where a top-down stream aims to predict stimuli, and a classifier configuration with inverted streams (Fig 1 b2). Cortex may implement either configuration; however, due to behavioral and neuroscience studies supporting top-down prediction in the cortex, we will focus on the generative configuration in this discussion.

#### 4.1.1. Explicit error and representation neurons.

Like in the original model by Rao and Ballard [29], errors are encoded in mismatch neurons, and predictions are encoded in a separate population of representation units. Following the canonical microcircuit for predictive coding, we hypothesize that the neuron types are pyramidal cells (PYR) found in the superficial (L2/3) and deep (L5) layers of cortex respectively [65,82]. These populations are directly measurable, and separate our model from recent hypotheses like dendritic hPC [25] or the dendritic cortical microcircuits of Sacramento et al. [30], which assume populations of representation neurons inhibited by local interneurons.

While error units can be recognized by their decreasing activity during learning, representation units are not as easily identified: due to the recurrent structure of our model, representation units may code a mixture of prediction and mismatch. The degree of mixture can be varied by the conductance couplings $\left\{g^{\text{rep}},g^{\text{err}}\right\}$ of the two compartments (see Section 4.3). It may be the case that *g*^err^ is much larger than *g*^rep^; then, representation units mostly encode errors during learnin*g*, while their activity is fully explained by predictions in absence of mismatch signals. Indeed, this reflects the recent analysis of Aceituno et al. [83], showing that in-vivo activity of L5 PYR better aligns with strong influence of error signals (‘target learning’).

#### 4.1.3. Different dynamics of prediction and error units encode their roles.

The two types of neurons in our model do not only encode different information, but also use different dynamics to perform their respective computations. In general, the somatic integration of dendritic activity involves a complex interplay of addition and multiplication [84]. In this work, algorithmic requirements lead us to assume purely multiplicative integration for error neurons, and purely additive integration for representation neurons. Mechanistically, this distinction might originate from the spatial organization of inputs onto the dendritic tree and/or the proximity of the compartment to the soma.

Our model predicts that neuronal outputs of L2/3 PYR are formed by modulation of bottom-up errors with the activity of same-area L5 representation units: the concrete prediction of the model is that error neurons are silenced if activity of representation units saturates, $e\propto (r^{r}{)}^{\prime }=\varphi^{\prime }({\overset{˘}{u}}^{\text{som}})$.

#### 4.1.4. Dendrite dynamics before and after learning.

In our model, the teaching signal from which representation units learn is encoded in an error-receiving dendritic compartment *u*^err^. Learning reduces this signal, until the ^pred^iction-receiving compartment *u*^pred^ is fully predictive of somatic activity (see Fig G in S1 Text, left). As shown in Fig 1 a1, the two compartments may be realized by separate apical, basal and perisomatic dendrites. See S1.8 in S1 Text for an in-depth discussion.

In a recent study by Francioni et al. [56], they find that the difference between somatic and distal (!) apical activity in L5 PYR correlates with mismatch signals, where they distinguished two populations integrating positive and negative errors respectively. Francioni et al. used a specific BCI task, and it remains to be studied whether their findings extend to more general task setups; due to the relevance of this experimental work for our model, we discuss it further in S1.4 in S1 Text.

#### 4.1.5. Inter-area connectivity.

**Fig 7:**
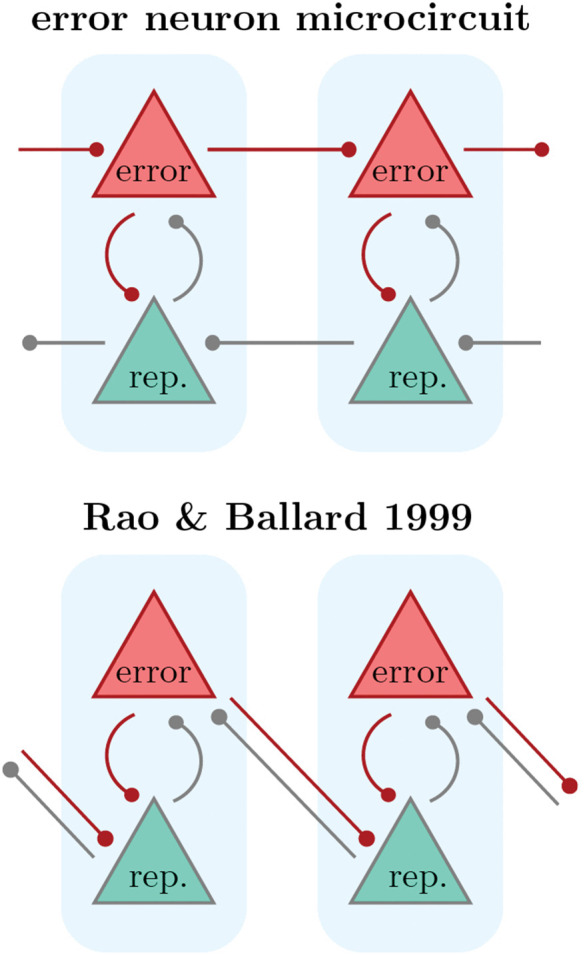
Connectivity in our model and classical PCNs.

Our model differs from classical PCNs (e.g., the Rao-Ballard model [29]) by its inter-area connectivity scheme, see Fig 7. Our configuration has multiple benefits in terms of computational power and biological plausibility of the neuronal calculations, as we describe in Section 4.2.

In detail, our model predicts two separated streams of errors and predictions. Various studies have investigated preferential targeting of dendritic compartments by top-down and bottom-up projections [14–16,42,55] and mapped out distinct feed-forward and feedback pathways in the cortical hierarchy [52].

Concretely, if we assume that representation units are PYR in L5, and errors are encoded in L2/3, this predicts a top-down stream of L5 → L5 projections, and an opposing error stream projecting L2/3 → L2/3. This prediction concerns the effective functional connectivity between areas, regardless of the underlying anatomical implementation (see S1.5 in S1 Text for an extended discussion on a recent experimental result).

Alternatively, our model can be modified such that representation units do not project locally to error units in the same area, but to error units in upstream areas; see S1.6 in S1 Text.

#### 4.1.5. Local connectivity.

As in the Rao-Ballard model, our model assumes same-area connectivity between error and prediction units, which may be implemented by L2/3 and L5 PYR. Local connections between these populations are well characterized, see [85] for a recent review.

L2/3 neurons receive strong projections from L4, which we interpret as the last step in a feedforward inter-area pathway of errors, in line with the input to *e*^err^ in our model. Additionally, L2/3 neurons (in particular L2) receive strong input from PYR of L5A [86–89], in line with the input to *e*^rep^ in our model.

Computationally more important however is the local connectivity of L2/3 → L5, as this communicates the error signal to representation units (at their dendritic compartment *u*^err^). Strong connectivity from L2/3 to L5 has been observed [85,90], and is part of the canonical cortical microcircuit [91,92]. Our model predicts that these projections are crucial for learning. For example, if the relevant L2/3 → L5 projections are suppressed, then L5 PYR only integrate top-down activity, and there is no differential local error signal from which these units can learn.

For all local connections, an exact implementation of the backpropagation algorithm indicates a one-to-one matching of representation and error units (perfectly columnar local connectivity). For plausibility, we have rather assumed a noisy version of one-to-one matching, effectively connecting all representation and error neurons more or less strongly, still with preferential targeting. More concretely, biology may implement an exponential distance rule of local axonal arbors, i.e., local connectivity *L*^RE^ and *L*^ER^ scaling as $\propto e^{-d_{ij}^{2}}$, where *d*_*ij*_ is the distance between neurons *i* and *j*, similarly to what has been observed in inter-area connectivity [93–95].

#### 4.1.6. Sensitivity to neuronal variability.

The results of Fig 6 a show that error neuron microcircuits are largely insensitive to variability of representation units, but variability on errors easily disrupts learning. We have identified that this is due to variability on the offset of error units (Fig C in S1 Text), changing the sign of error signals. Algorithmically, this makes sense: it is known that successful learning requires errors of the correct sign, whereas the magnitude is not as important (see same-sign FA [77,96]). Therefore, our model makes the experimental prediction that perturbing error units disrupts learning much more than perturbing of representation units, which can be tested by injecting the same noise levels to L2/3 and L5 neurons respectively.

### 4.2. Embedding into cortical models of learning and predictive processing

Most implementations of error learning in the cerebral cortex can be grouped into two categories: those with error neurons (as in the classical Rao-Ballard model [29]), and models where errors are constructed locally on the dendrites of representation units [25,30]. As of now, dendritic error construction is not well tested as a model of the cortical hierarchy, and scaling to multi-area networks remains to be proven. In [30], Sacramento et al. successfully trained a three-area network on the MNIST digit classification task; however, the same performance can be reached by leaving out the first area [97], making it unclear if useful error signals have reached the early area in these results.

In this work, we show that explicit error neurons facilitate more stable learning across cortical areas, as dendritic error construction is susceptible to parameter variability, caused by imperfect lateral inhibition. In short: explicit error neurons allow errors to be propagated through the network *and then* sent to representation units, while dendritic error construction (like classical predictive coding) requires the calculation of a difference of activity *in every area*. It is precisely this difference which is propagated as the error; under imperfect weights, this reconstruction of useful error signal fails, as Fig 4 a shows. Another important advantage of explicit error neurons is their increased computational power, as reflected by the ability to solve the spatio-*temporal* credit assignment problem by approximating backpropagation-through-time (BPTT); see Section 4.3 and [44].

### 4.3. How closely do we need to implement ‘backpropagation and the brain’?

BP is the algorithm underlying the deep learning revolution, and in practice currently the only way ANNs are trained to solve complex tasks. This is because BP propagates local errors across all areas, tailored to each neuron according to its contribution to task performance. While it is clear that cortex does not implement the error backpropagation algorithm *exactly* as it is formulated in machine learning, one may explore the hypothesis that some approximate version can explain learning [1,2]. In our model, backpropagation is demonstrably obtained in the limit $g^{\text{err}}\to 0$, where error and prediction pathways decouple, in conjunction with strictly hierarchical connectivity and identity local weights (Section 6.2). While the inclusion of such deviations from an ‘ideal’ implementation of BP is motivated by biological plausibility, it is important that such properties can also be interpreted as features, e.g., as implementations of advanced machine learning techniques. Thus, we now discuss how both non-hierarchical connectivity and non-weak nudging ($g^{\text{err}}\text{⧸}\to 0$) can fit together with backpropagation on a firm theoretical understanding.

#### 4.3.1. Loose hierarchy and recurrency.

Following connectivity in the Macaque visual cortex, we have organized connections in our model into preferential hierarchical streams, but also include skip connections. More generally, other modalities may implement less hierarchical organization, as non-primate species do in general [95,98–100]. At first glance, this seems at odds with the success of hierarchically organized ANNs, and one may assume that this excludes any comparison between feed-forward ANNs and biological networks. Algorithmically however, skip connections can be made sense of through their relation to residual networks [101,102], which draw their computational power from the central motif of identity (skip) projections.

Note however that the vanilla BP algorithm is derived for strictly hierarchical networks only (layered graphs), where the activity of each neuron is causally dependent only on input from earlier areas, and can be calculated in a closed form. This can be extended to include skip connections (directed acyclic graphs), as we do in this work. However, for general, recurrent architectures including loops and self-recurrency, one needs to take the dependence of each neuron on past network states into account. To do so, one can generalize BP to the BPTT algorithm. This solves the issue by unrolling the recurrent network in time to obtain a layered graph, increasing however biological implausibility. Online learning approximations to BPTT have been proposed [44,79,103,104], and future work may investigate microcircuit adaptations of these algorithms. In particular, the solution in [44] incorporates the same representation and error neuron populations presented in this work, thus lending itself to a cortical microcircuit implementation.

#### 4.3.2. Non-weak nudging.

While classical implementations of PC require representation units in the first area to be clamped to the stimulus, our model allows for a more organic introduction of targets into the network. Instead of clamping, it couples error and representation units with a conductance parameter *g*^err^, with the ability to implement both weak nudging ($g^{\text{err}}\to 0$) [105] and strong nudging, restoring clamping in the limit of very large *g*^err^. As a result, representation units across areas encode a mixture of bottom-up errors and top-down representations. This has important implications for experimental predictions: in a strict interpretation of ‘backpropagation and the brain’, one expects to find that representation units do not encode errors at all. This be*g*s the question of how representation units can access error information in order to update their weights, and is at odds with recent experimental evidence by Aceituno et al. [83]. In fact, the results of [83] suggest that representation units are strongly influenced by targets during learning, aligning with the clamping hypothesis. In our setting, varying the conductance *g*^err^ provides an organic way to shift from backprop-like (weak nud*g*ing [37,105]) to target-like learning (clamping).

Coincidentally, theoretical work on bio-plausible backpropagation has demonstrated successful training on larger datasets even for strong nudging [38,106,107]. Intuitively, by changing the influence of errors on the network, representation units change the relative mixture of how strongly they encode bottom-up and top-down information, and weights may follow a completely different trajectory than that of strict backpropagation; we observe this effect in our model in Fig A b (see S1 Text), where networks train well even as their weight updates differ up to 90° from backpropagation.

In conclusion, this demonstrates how advanced machine learning techniques can inform models of learning and connectivity, and shows that ‘backpropagation in the brain’ implies a landscape of theories with distinct degrees of bio-plausibility.

### 4.4. Future work

Several issues remain unaddressed in this work, some of which we have highlighted in the previous sections. Notably, we have tested our model on supervised benchmarks, which require external target labels. However, by replacing the loss function, our model can also accommodate other learning schemes, where latent representations are formed purely by observation, or only sparse rewards are provided.

Aditionally, our model retains limitations in terms of biological plausibility. First, our error units have linear activations, i.e., they can produce both excitatory and inhibitory outputs. This issue has been discussed in the literature, with a range of possible solutions based on separate populations for negative and positive errors [65,66], error signals coded with respect to a bias, or transformed errors [66–70]. Second, synapses in our model can change their weights from excitatory to inhibitory, and vice versa, in contradiction with biology (Dale’s law). Recent works have successfully trained networks of purely excitatory and inhibitory populations [108,109], but rely on standard training libraries without a mechanistic explanation of the error propagation. It remains an open question how to model error neuron microcircuits observing Dale’s law. Third, our model remains a rate-based model. An extension to spikes can be conceived, guided by a recent spiking implementation [78] of the dendritic error construction model [30].

Further, our model singles out the interconnectivity of feedback-projecting PYR in L5 and feed-forward-projecting L2/3, excluding other cell types and projections both within the cortex and with other brain structures. While this is motivated by the high degree of connectivity between L5 and L2/3, incorporating other known projections into our model implementing the algorithmic core of backpropagation could lead to insights. Particularly relevant projections absent from our model but abundant in biology are lateral (recurrent) projections between PYR of the same layer. Thalamo-cortical projections from higher-order thalamic nuclei also seem to be particularly pertinent, given their strong influence on L5 and L1 [110] and their computational relevance [111–116]. Finally, our work doesn’t account for the full complexity of neuronal and in particular dendritic temporal dynamics. Future work may investigate fast top-down prediction and slower error signaling, which produce distinct time scales in experimental signatures [108].

## 5. Conclusion

The ‘backpropagation and the brain’ hypothesis remains controversial, but as we have argued, the discovery of error units in cortex and the assumption that the brain uses existing errors to their fullest makes it a compelling theory to study. An important milestone in this regard is the realization that predictive coding can be formulated as an approximation of backpropagation along arbitrary computational graphs [21–24].

Our model aims at filling the gap between deep learning and neuroscience: Its novelty lies in combining a bio-plausible, multi-compartment neuron model with the scalable architecture and efficient functional learning of BP. Further, we have relaxed the one-to-one matching of error and representation units, and implement bio-realistic connectivity based on the Macaque visual cortex [40].

By its construction, our model harnesses the proven functionality of ANNs (where deep learning is established as the state of the art), and extracts connectivity and microcircuit motifs which guide a bio-plausible model. This top-down approach separates our model from bottom-up approaches (e.g., E-I balanced networks or Gabor filter models), which are difficult to functionalize [1,117] or rely on unexplained, external learning mechanisms such as the surrogate gradient method.

In this work, we have shown that the error neuron microcircuit model can closely approximate the backpropagation algorithm, and that such networks perform well and scale to solve complex tasks requiring learning across multiple areas. This provides the basis for future work to clarify whether, and to which degree, the brain implements a form of error backpropagation.

## 6. Methods

### 6.1. Bio-plausibility of the error backpropagation algorithm

In a hierarchical ANN with ℓ=1,...,N layers, representations are projected through weights $W_{\ell ,\ell -1}$, and layer-wise errors $\delta_{\ell }$ can be obtained recursively from a target provided to the output layer *N*. The error backpropagation algorithm, applied to such an ANN learning a non-temporal task, consists of two computational steps: the backpropagation of errors,


$$\delta_{\ell }=\varphi^{\prime }(u_{\ell })⊙[W_{\ell +1,\ell }{]}^{T}\;\delta_{\ell +1}\;,$$ (6)

and the weight update rule


$$\Delta W_{\ell ,\ell -1}=\delta_{\ell }\;r_{\ell -1}^{T}\;.$$ (7)

Implementing these computations into a bio-plausible model carries several obstacles [1,2]. Our model solves the most pertinent ones in the following way, see Table 1.

### 6.2. Neuronal dynamics and learning rule

Our microcircuit model implements Equations 6 and 7 in the following way. We define a network with ℓ=1,...,N areas, equivalent to layers in the context of ANNs. In our model, we relax the strict hierarchical connectivity, and allow for connections between all areas.

The soma dynamics of the population of representation units in area ℓ, written as a vector $u_{\ell }^{\text{som}}$, are


$$C_{m}\frac{\text{d}}{\text{dt}}u_{\ell }^{\text{som}}=-g_{\text{l}}u_{\ell }^{\text{som}}-g^{\text{rep}}(u_{\ell }^{\text{som}}-u_{\ell }^{\text{pred}})-g^{\text{err}}(u_{\ell }^{\text{som}}-u_{\ell }^{\text{err}})\;,$$ (8)

that is, a leaky integration of the prediction- and error-receiving compartments’ voltages $u_{\ell }^{\text{pred}}$ and $u_{\ell }^{\text{err}}$. Compartments are modeled as


$$u_{\ell }^{\text{pred}}=\sum_{k\neq \ell }W_{\ell ,k}\;r_{k}^{r}\;u_{\ell }^{\text{err}}=L_{\ell ,\ell }^{\text{RE}}r_{\ell }^{\text{E}}\;,$$ (9)

where $W_{\ell ,k}$ represents the matrix of weights connecting area *k* to ℓ. $u_{\ell }^{\text{pred}}$ sums all inputs from representation units in other areas (but not from the same area), whereas $u_{\ell }^{\text{err}}$ is formed from output of error neurons in the same area.

The neuronal output is the ‘prospective’ rate [43]


$$r_{\ell }^{r}=\varphi ({\overset{˘}{u}}_{\ell }^{\text{som}})=\varphi (u_{\ell }^{\text{som}}+\tau_{r}^{r}\;\frac{\text{d}}{\text{dt}}u_{\ell }^{\text{som}})$$ (10)

with $\tau_{r}^{r}$ a neuron-wise parameter, called the prospective time constant. For quasi-instantaneous transmission, we set $\tau_{r}^{r}=\tau_{m}^{r}\equiv \frac{C_{m}}{g_{\text{l}}+g^{\text{rep}}+g^{\text{err}}}$. For tasks requiring memory, we encode short-term memory through slow neuron responses, for which $\tau_{r}^{r}<\tau_{m}^{r}$ (see Section 6.5 and 6.6).

Error units follow the dynamics


$$C_{m}\frac{\text{d}}{\text{dt}}e_{\ell }^{\text{som}}=-g_{\text{l}}e_{\ell }^{\text{som}}-g^{\text{den}}(e_{\ell }^{\text{som}}-e_{\ell }^{\text{rep}}⊙e_{\ell }^{\text{err}})\;,$$ (11)

with


$$e_{\ell }^{\text{rep}}⊙e_{\ell }^{\text{err}}=[L_{\ell ,\ell }^{\text{ER}}\;\varphi^{\prime }({\overset{˘}{u}}_{\ell }^{\text{som}})]⊙\sum_{m}B_{\ell ,m}\;r_{m}^{\text{E}}.$$ (12)

Note that $e_{\ell }^{\text{rep}}=[L_{\ell ,\ell }^{\text{ER}}\;\varphi^{\prime }({\overset{˘}{u}}_{\ell }^{\text{som}})]$ does not carry the units of a voltage, but rather $\frac{\text{weight}\times \text{rate}}{\text{voltage}}=\frac{\text{voltage}}{\text{voltage}}=1$, i.e., it is unitless. Thus, the summand $g^{\text{den}}e_{\ell }^{\text{rep}}⊙e_{\ell }^{\text{err}}$ in Equation 11 is a current, and can be added to the somatic integration.

For learning, we need to inject a target signal into the network. We do so in the form of a target rate *r*^tgt^, which is provided to area *N* (‘output layer’) following slightly modified dynamics:


$$C_{m}\frac{\text{d}}{\text{dt}}e_{N}^{\text{som}}=-g_{\text{l}}e_{N}^{\text{som}}-g^{\text{nudge}}\;(e_{N}^{\text{som}}-L_{N,N}^{\text{ER}}\;\varphi^{\prime }({\overset{˘}{u}}_{N}^{\text{som}})⊙[r^{\text{tgt}}-r_{N}^{r}]).$$ (13)

This means that the representation neurons in area *N* communicate their rate $r_{N}^{r}$ locally to error units, which calculate the difference between *r*^tgt^ and $r_{N}^{r}$. Error neurons also implement prospective rates,


$$r_{\ell }^{\text{E}}=\varphi ({\overset{˘}{e}}_{\ell }^{\text{som}})=\varphi (e_{\ell }^{\text{som}}+\tau_{r}^{\text{E}}\;\frac{\text{d}}{\text{dt}}e_{\ell }^{\text{som}})\;,$$ (14)

and we set all error neurons to be instantaneous, with $\tau_{r}^{\text{E}}=\tau_{m}^{\text{E}}=\frac{C_{m}}{g_{\text{l}}+g^{\text{den}}}$ for hidden areas; $\tau_{r}^{\text{E}}=\tau_{m}^{\text{E}}=\frac{C_{m}}{g_{\text{l}}+g^{\text{nudge}}}$ for the output area. Note that in all of our simulations, we choose a linear activation for error units, $\varphi ({\overset{˘}{e}}_{\ell }^{\text{som}})={\overset{˘}{e}}_{\ell }^{\text{som}}$.

By plugging Equation 12 into Equation 11, we can see that ${\overset{˘}{e}}_{\ell }^{\text{som}}$ encodes the error in each area:


$$\begin{array}{cc} {\overset{˘}{e}}_{\ell }^{\text{som}} & =\frac{g^{\text{den}}}{g_{\text{l}}+g^{\text{den}}}[L_{\ell ,\ell }^{\text{ER}}\;\varphi^{\prime }({\overset{˘}{u}}_{\ell }^{\text{som}})]⊙\sum_{m}B_{\ell ,m}\;{\overset{˘}{e}}_{m}^{\text{som}}\;,\\ {\overset{˘}{e}}_{N}^{\text{som}} & =\frac{g^{\text{nudge}}}{g_{\text{l}}+g^{\text{nudge}}}\;L_{N,N}^{\text{ER}}\;\varphi^{\prime }({\overset{˘}{u}}_{N}^{\text{som}})⊙[r^{\text{tgt}}-r_{N}^{r}]\;. \end{array}$$ (15)

In a hierarchical setup with ideal local weights, $L_{\ell ,\ell }^{\text{ER}}=1$, this implements Equation 6, under the assumption that ${\overset{˘}{u}}_{\ell }^{\text{som}}=u_{\ell }^{\text{pred}}$ (see below).

The dynamics of the representation-projecting weights $W_{\ell ,k}$ are given by


$$\frac{\text{d}}{\text{dt}}W_{\ell ,k}=[{\overset{˘}{u}}_{\ell }^{\text{som}}-\frac{g^{\text{rep}}}{g_{\text{l}}+g^{\text{rep}}+g^{\text{err}}}u_{\ell }^{\text{pred}}]\times r_{k}^{r}\;,$$ (16)

where by comparison with Equation 4 we can identify the total conductance as $g^{\text{tot}}\equiv g_{\text{l}}+g^{\text{rep}}+g^{\text{err}}$. By rewriting ${\overset{˘}{u}}_{\ell }^{\text{som}}=\frac{g^{\text{rep}}\;u_{\ell }^{\text{pred}}+g^{\text{err}}\;u_{\ell }^{\text{err}}}{g_{\text{l}}+g^{\text{rep}}+g^{\text{err}}}$, we obtain


$$\begin{array}{cc} \frac{\text{d}}{\text{dt}}W_{\ell ,k} & =\frac{g^{\text{err}}}{g_{\text{l}}+g^{\text{rep}}+g^{\text{err}}}\;u_{\ell }^{\text{err}}\times r_{k}^{r}\\ & =\frac{g^{\text{err}}}{g_{\text{l}}+g^{\text{rep}}+g^{\text{err}}}\;L_{\ell ,\ell }^{\text{RE}}\;r_{\ell }^{\text{E}}\times r_{k}^{r}\\ & =\frac{g^{\text{err}}}{g_{\text{l}}+g^{\text{rep}}+g^{\text{err}}}\;L_{\ell ,\ell }^{\text{RE}}\;{\overset{˘}{e}}_{\ell }^{\text{som}}\times r_{k}^{r}\;. \end{array}$$ (17)

For identity local weights and hierarchical connectivity, we find that this implements Equation 7.

However, there is one additional caveat to the exact correspondence of our model and backpropagation: due to the coupled compartments, the soma of representation units does not only encode external inputs, ${\overset{˘}{u}}_{\ell }^{\text{som}}\neq W_{\ell ,\ell -1}r_{\ell -1}^{r}$. Instead, errors are added to somata in every area, confounding the activity of all units as a combination of inputs and errors due to the recurrency in the network. BP is only strictly equivalent to our model and predictive coding implementations under the ‘fixed prediction’ assumption, i.e., where the feed-forward activity ${\overset{˘}{u}}_{\ell }^{\text{som}}=W_{\ell ,\ell -1}r_{\ell -1}^{r}$ needs to be saved to be used during learning.

This is reflected in the angle of alignment between ΔW in ANNs and our model (cf. Fig 4 b), which tends to be larger than zero. This can be ‘remedied’ by decreasing the influence of the error-receiving compartment on the soma, $g^{\text{err}}\to 0$, a limit also known as weak nudging [105]. However, this also shrinks the learning signal, as evident from Equation 17, which in turn leads to higher variability sensitivity and is less faithful to tentative in-vivo evidence [83]. Instead, we keep *g*^err^ as a free parameter; as our results show, our model performs on par with an ANN even for large nudging parameters, implyin*g* that neuron activities do not need to exactly follow those of feed-forward ANNs for successful learning.

### 6.3. Network connectivity

#### 6.3.1. Visual cortex connectivity.

To model realistic inter-area connectivity, we use the weighted connectivity matrix determined by Markov et al. 2014 [40] (Fig 1 b1 therein, first six areas reproduced in Fig 1 b1), measured by retrograde tracing.

To model the learning of a new task based on an existing network structure, we initialize each network using the relative connectivity strength found in Fig 1 b1. For example, weights between V2 → V4 are larger than those between V2 → TEO by a factor of about 3 at initialization. We round all projections below 10^–2^ down to zero, and set the diagonal of Fig 1 b1 to zero, as the recurrency within an area is already encoded in the local connectivity motif (local communication between representation and error units).

#### 6.3.2. Weight symmetry.

In our networks, errors are projected through weights $B_{\ell ,m}$. Exact error backpropagation requires symmetric weights between two areas, i.e., $B_{\ell ,m}=[W_{m,\ell }\;{]}^{T}$. While networks can learn even with non-symmetric, static feedback weights (Feedback Alignment, FA [73]), this has been shown to not scale well to complex problems, requiring more neurons to successfully learn a task [74,96,118–120].

An efficient mechanism to learn symmetric weights between two areas is Phaseless Alignment Learning (PAL) [74]. PAL uses temporal noise as an information carrier, allowing to simultaneously learn all error projection weights $B_{\ell ,m}$, without disruption of representation unit weights $W_{m,\ell }$. Note that PAL makes use of the same principles as the mechanism for local reconstruction of $\varphi^{\prime }$ proposed in this work, which we explain in Section 6.4.

In [74], we have shown that PAL is able to efficiently train the dendritic microcircuit model of Sacramento et al. [30]; however, its simulation on conventional hardware is time-consuming due to the requirement of fine-grained simulations of temporal noise.

For computational feasibility, we therefore approximate PAL by setting error projecting weights to the transpose of representation unit weights, and adding noise to mimic the approximate degree of alignment typically found in networks using PAL. We also need to consider that the numbers of error and representation units are not required to be equal in our network. To account for this, we pad the weight matrix with zeros: for example, $1_{\ell ,\ell }^{\text{RE}}$ is an *n*_*R*_ × *n*_*E*_ matrix (*n*_*R*_: number of representation units in area ℓ; *n*_*E*_: number of error units), with the ‘diagonal’ entries set to 1, and all others set to 0. For example, for *n*_*R*_ = 2 and *n*_*E*_ = 3 in area ℓ, we have


$$\begin{array}{c} 1_{\ell ,\ell }^{\text{RE}}=(\begin{array}{ccc} 1 & 0 & 0\\ 0 & 1 & 0 \end{array}) \end{array}$$

After every time step, error projections are set to $B_{\ell ,m}=[1_{m,m}^{\text{ER}}\;W_{m,\ell }\;1_{\ell ,\ell }^{\text{RE}}\;{]}^{T}+\Xi_{\ell ,m}$, with $\Xi_{\ell ,m}\sim 𝒰(-0.5,0.5)$ sampled *once* (i.e., at the initialization of the network).

#### 6.3.3. Local connectivity.

In our model, we relax the strict one-to-one matching of error and representation units found in many models [24,29,30,43,121]. To model noisy preferential targeting between representation and error units, we fix local weights to $L_{\ell ,\ell }^{\text{RE}}=1_{\ell ,\ell }^{\text{RE}}+\Xi_{\ell ,\ell }$, with $\Xi_{\ell ,\ell }\sim 𝒰(-\sigma_{L},\sigma_{L})$, and similarly for $L_{m,m}^{\text{ER}}$. See Section 6.5 and 6.6 for all simulation parameters.

### 6.4. Non-linear dendrites enable local computation of $\varphi^{\prime }(u)$ in error units

Here, we explain how the error neurons reconstruct $\varphi^{\prime }(u)=\frac{\partial \varphi (u)}{\partial u}$ in their dendritic compartment. To simplify notation, we use a generic voltage *u* in this subsection, acting as a stand-in for the prospective voltage ${\overset{˘}{u}}^{\text{som}}$ of the representation units.

Error neurons are able to estimate $\varphi^{\prime }(u)$ using biologically plausible ingredients: temporal noise, which is ubiquitous in the brain, and simple functions (high-pass filtering, gating and temporal averaging) that a dendrite can perform.

To start, we take a local population of representation neurons with membrane potential vector *u*, and add noise fluctuations for each neuron, u+ξ, where ξ can be, e.g., white noise; note that any noise with sufficiently short correlation time suffices. In order for the neurons to encode a useful representation, the signal-to-noise ratio needs to sufficiently high, meaning that |u|≫|ξ|. Coincidentally, this is a useful property which we exploit in the following:

The error neuron receives the noisy output of the representation unit at its dendrite,


$$r=\varphi (u+\xi )\approx \varphi (u)+\xi ⊙\varphi^{\prime }(u)$$ (18)

where we have expanded the rate to first order in small noise ξ. Next, we aim to isolate the component of *r* governed by noise (second term). Note that *u* is driven by stimuli and error signals in the network, which are of low frequency compared to the fast noise ξ. Therefore, we hypothesize that the error neuron dendrites integrate a high-pass filter, leading to


$$\hat{r}=\xi ⊙\varphi^{\prime }(u)\;.$$ (19)

By applying non-linear gating onto its input, e.g., in the form of a rectified linear (relu) function, and averaging over noise samples, the dendrites compute


$$\langle \;\text{relu}\;(\hat{r})\;\rangle \propto \varphi^{\prime }(u)\;.$$ (20)

**Fig 8:**
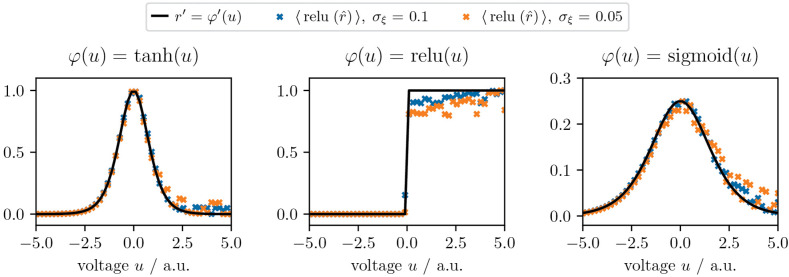
Temporal noise and non-linear dendrites enable the reconstruction of $\varphi^{\prime }(u)$ in error neurons. A representation unit encodes a signal voltage *u* for *T*_pres_ = 100 dt, and white noise $\xi \sim 𝒩(0,\sigma_{\xi })$ is sampled at every dt. After high-pass filtering with $\tau_{\xi }=10\text{ dt }$, the mean dendritic activity accurately tracks $\varphi^{\prime }(u)$ for different activation functions. The overall normalization of each curve has been rescaled for ease of illustration.

This shows that a rescaled version of $\varphi^{\prime }(u)$ can be computed locally in the error neuron dendrite, for sufficiently smooth activation functions. See Fig 8 for simulation results for one pair of representation and error units.

### 6.5. Simulations

We simulated the error neuron microcircuits using the Euler forward method with discrete time steps dt, implementing the neuronal dynamics of Equations 8, 9, 10, 12, 11, 13, and 14 and the plasticity of Equation 16. Note that neuronal and weight dynamics are applied during all time steps, without phases or information buffering. The implementation builds on the code of [74], simulating the model of dendritic error microcircuits [30], see Methods in [74] for details. In all experiments, all weight matrices are fully connected, and we allow voltages to settle during a brief settling phase of several dt. Data is fed into the networks as pairs of input and targets (supervised training), where each data point is presented for *T*_pres_ time steps. As opposed to the model of Sacramento et al., our microcircuit takes target rates *r*^tgt^ instead of voltages (Equation 13, see also below).

For parameter values, see Section 6.6.

#### 6.5.1. Cart pole and DMS task.

For the cart pole task, we trained our error neuron microcircuit networks to reproduce the control properties of the linear-quadratic regulator (LQR). We randomly sampled static input vectors $\left[x,\dot{x},\theta ,\dot{\theta }]\sim 𝒰(-1.5,1.5\right)$ and recorded the ouput of the LQR as the teaching signal.

For validation and testing, we use a simulation of the non-linear dynamics of a cart pole, with the error neuron microcircuits acting as a controller. Note that the time step width of the cart pole simulation is equivalent to that of the microcircuits. For every time step dt, the controller receives the vector $\left\{x,\dot{x},\theta ,\dot{\theta }\right\}$ as input and produces a scalar controlling signal *F*, which is passed to the physical cart pole simulation. The cart pole dynamics are then advanced by one step, closing the loop between controller and system. The cart pole is initialized with all variables $\left\{x,\dot{x},\theta ,\dot{\theta }\right\}$ sampled uniformly from 𝒰(−1,1). For the angles, this corresponds to approx. $\pm \;57^{\circ }$. The cart pole is reset if |θ| exceeds 70°. Test performance is measured by the penalty log, i.e., how many time steps ($\text{dt}=10^{-2}$ ms) the cart pole is lying per epoch (20 s).

For the DMS task, we set up a network of two hidden and one output representation neurons and an equal number of error neurons. The task is constructed from train, validation and test sets of 50 stimuli pairs ({↑↑,↑↓,↓↑,↓↓}) together with the corresponding target ({↑,↓,↓,↑}). The input data is a 1-dim. time series consisting of an initial fixation period of 250 ms, a first stimulus phase of length 250 ms, a delay period of random length ~𝒰(50,250), and the second stimulus presented for 250 ms. Only at the last step of this time series, the network is provided with the target.

To encode memory at a useful timescale, we chose the conductances of the representation neurons to accommodate an effective membrane time constant of $\tau_{m}^{r}\approx 2.2\;\text{dt}=220$ ms. In order to propagate the effects of memory across the rate level to downstream areas, we use retrospective representation neurons [44], for which $\tau_{m}^{r}=0$. Error neurons are quasi-instantaneous, $\tau_{m}^{\text{E}}=\tau_{r}^{\text{E}}$. For the activation function of output neurons, we picked 20×tanh(x−0.5), representing a smooth binary classification function.

As a control, we simulate a layered RNN with an equivalent architecture and dynamics, two hidden and one output neuron. Each neuron is self-recurrently connected, implementing leaky integration of bottom-up inputs (input → hidden, hidden → output; there are no other recurrent connections). The timescale of the self-recurrent connections is set equivalent to $\tau_{m}^{r}$, and the activation functions are the same as in the error circuit. We trained the network with BPTT and report the accuracy in Fig 2 b2.

#### 6.5.2. Yin-Yang task.

For this task, we simulated networks of size [30–3] with four inputs for each data point (encoding of position through *x*, *y*, (1 − *x*) and (1 − *y*)), following [76]. The results for the dendritic error construction model (Sacramento et al., 2018) are reproduced from [74]. Note that the results labeled ‘Sacramento et al., 2018 (FA)’ use random, fixed backprojections (feedback alignment). Additionally, for the ‘Sacramento et al., 2018 (PAL)’ runs, PAL was fully simulated, instead of approximated; whereas all other experiments in this work use approximate weight transport (*B* = *W*^*T*^ + noise).

#### 6.5.3. Teacher-student experiments.

The experiments in Fig 4 are designed to enable a fair comparison with the models of [30] and [25]. To do so, we generate a synthetic dataset by feeding random inputs to a teacher ANN with randomly initialized, fixed weights, and recording the output of the teacher network. Inputs and weights are sampled such that the input-output mapping is non-trivial (that is, non-linear).

To demonstrate that our model scales to train many cortical areas, we increase the task difficulty by scaling the architecture of the teacher and student networks from [2–1] neurons (no hidden layer) to [4–2-1], [8–4-2-1], [16–8-4-2-1] and finally [32–16-8-4-2-1] neurons (3 hidden layers). Neuron numbers double in order to make training of all areas necessary, such that errors actually need to be backpropagated, instead of a relying on learning only of output area weights. The dataset is then fed into our network in the classification configuration, such that representations propagate downstream to match the teacher output.

We also train the model of Sacramento et al. [30], using the implementation described in [74]. To enable a fair comparison, we augment the model with the prospective coding mechanism, and initialize the model with ideal lateral weights $W_{k,k}^{\text{IP}}$ and $W_{k,k}^{\text{PI}}$ (‘self-predicting state’). In order to facilitate tight matching of lateral inhibition, we set the learning rates of $W_{k,k}^{\text{IP}}$ to be twice that of the efferent feed-forward weights $W_{k+1,k}^{\text{PP}}$. The top-down weights $W_{k,k+1}^{\text{PP}}$ connecting pyramidal cells are adjusted in the same way as in our model, setting them to the transpose of forward-weights $W_{k+1,k}^{\text{PP}}$ plus noise (Section 6.3). The matching lateral weights are set to the noiseless (!) transpose after every time step, i.e., $W_{k,k}^{\text{PI}}=-[W_{k+1,k}^{\text{PP}}{]}^{T}$. We include this ideal weight transfer in order to more objectively represent the computational power of the model by Sacramento et al.

Furthermore, their model takes voltages $u_{N}^{\text{trgt}}$ as targets; in order to compare all models on the same task, we thus feed the teacher output *r*^tgt^ into the model as the voltage $u_{N}^{\text{trgt}}$. For a fair comparison, the MSE loss is then calculated based on the somatic prospective *voltage* ${\overset{˘}{u}}_{N}^{\text{P}}$ in the output area instead of the rate. Note that it is still the rate $\phi ({\overset{˘}{u}}_{N}^{\text{P}})$ which is propagated to lower areas.

Finally, we also train the model of Mikulasch et al. [25] in the following configuration: inputs are presented to the top-most area, and top-down predictions are propagated to the lowest level, which receives the target *r*^tgt^. We have implemented two non-linear variants of dendritic hPC, but observe no difference in performance; we thus have included the linear model in Fig 4. As described in [25], the computations performed in the model by Mikulasch et al. and that of Sacramento et al. are related. We relate them in detail in S1.2 in S1 Text. Compared to the model of Sacramento et al., our implementation of dendritic hPC abstracts away the interneurons, by setting the interneuron voltage to the rate of the lateral pyramidal cells, $u_{k}^{\text{I}}=r_{k}^{\text{P}}$; like the representation units, all interneurons have linear activation functions. In all other details, our simulations of dendritic hPC follow the same steps as described above for the model by Sacramento et al., see S1.2 in S1 Text.

To compute the angle between weight updates $∠(\Delta W,\Delta W^{\text{ANN}})$, we computed ΔW for each model (ANN, error neuron microcircuit, Sacramento et al., dendritic hPC) for each input-target pair of the test set, and calculate the cosine similarity between $\Delta W^{\text{ANN}}$ and the other models. In order to compare the error neuron microcircuit with skip connections to the hierarchical ANN, we only include the ΔW of neighboring areas in the angle calculation.

#### 6.5.4. Image generation tasks.

For the generative tasks, we selected one example image per digit class from the MNIST dataset, and trained the network on the pairs of classes and pixels. Image generation is fully deterministic; each class always produces the same image. There are no separate train and test sets in this task.

To model neuron variability (Fig 6 a), we initialized each neuron individually with an activation function with slope $\sim 𝒩(1,\sigma_{\text{act}})$ and offset $\sim 𝒩(0,\sigma_{\text{act}})$.

### 6.6. Simulations parameters

For all simulations, we set the resting potential to *E*_l_ = 0. We set the capacitance as *C*_m_ = 1, thus conductances have units ms^–1^. All networks (error neuron microcircuit, ANN, Sacramento et al. and dendritic hPC) are trained with vanilla gradient descent with batch size 1. All simulations were performed with 10 different random seeds. For all parameters, see Tables 2 and 3.

**Table 2:** Parameters for microcircuit model simulations.

	cart pole (Fig 2 a)	DMS (Fig 2 b)	multi-area (Fig 4)
dt [ms]	10^–2^	10^–2^	10^–2^
*T*_pres_ [ms]	1	800–1000	0.2
*g*_l_ [ms^–1^]	0.03	0.3⋆	0.03
*g*^rep^ [ms^–1^]	0.1	0.1	0.1
*g*^err^ [ms^–1^]	$6\times 10^{-4}{\;}^{•}$	0.06	0.06
*g*^den^ [ms^–1^]	0.1	0.1	0.1
*g*^nudge^ [ms^–1^]	0.06	0.06	0.06
$\tau_{r}^{r}$ [ms]	$\tau_{m}^{r}$	0	$\tau_{m}^{r}$
$\tau_{r}^{\text{E}}$ [ms]	$\tau_{m}^{\text{E}}$	$\tau_{m}^{\text{E}}$	$\tau_{m}^{\text{E}}$
input	$𝒰(-1.5,1.5{)}^{♣}$	DMS	𝒰(0,1)
target	teacher LQR♣	DMS	teacher ANN
train/val/test size	500/cart pole (20 s)	50/50/50	100/100/100
epochs	5	10	1 000
rep. units	[4,1]	[2,1]	variable^◊^
activation *φ*	tanh^♡^	[softplus(*x*, *β* = 10)^†^,	tanh
		20 × tanh(*x* − 0.5)]	
error units	[4,1]	[2,1]	as rep. units
weight init $W_{\ell ,\ell -1}$	$𝒰(-1,1{)}^{♠}$	𝒰(−1,1)	$𝒰(-1,1{)}^{♠}$
local weight $\sigma_{L}$	3^♡^	0.3	0.3
noise on *B* = *W*^*T*^	𝒰(−0.5,0.5)	𝒰(−0.5,0.5	𝒰(−0.5,0.5)

• Nudging strength scaled down to decrease learning speed; the same results are obtained by reducing the learning rate, cf. Equation 17.

⋆ Leak increased to obtain larger $\tau_{m}^{r}\approx 220$ ms, facilitating longer memory.

♣ Cart pole networks are trained on the input-output mapping of the LQR (teacher-student task).

♡ For the ideal microcircuit, we set the activations to be linear and $\sigma_{L}=0$.

♠ Teacher ANNs are initialized with weights $W_{\ell +1,\ell }\sim 𝒰(-1,1)$ as well.

♢ Area sizes increase with task complexity, see Section 6.5.

^†^ Softplus$\left(x,\beta )=\frac{1}{\beta }\log(1+\exp(\beta \cdot x)\right)$

**Table 3:** Parameters for microcircuit model simulations (cont’d).

	generative MNIST task (Figs 5 and 6)
dt [ms]	10^–2^
*T*_pres_ [ms]	0.2
*g*_l_ [ms^–1^]	0.03
*g*^rep^ [ms^–1^]	0.1
*g*^err^ [ms^–1^]	0.06
*g*^den^ [ms^–1^]	0.1
*g*^nudge^ [ms^–1^]	0.06
$\tau_{r}^{r}$ [ms]	$\tau_{m}^{r}$
$\tau_{r}^{\text{E}}$ [ms]	$\tau_{m}^{\text{E}}$
input	integers [0,...,9] as classes
target	MNIST pixels (28 × 28), one for each class
train/val/test size	10/10/10‡
epochs	1000
rep. units	[784, *n*_*R*_] (Fig 5), [784, 1000] (Fig 6 a), [784, 500, 500] (Fig 6 b)
activation	$\frac{1}{1+\exp(-x)}$
error units φ	[784, *n*_*E*_] (Fig 5), [784, 1000] (Fig 6 a), [784, 500, 500] (Fig 6 b)
weight init $W_{\ell ,\ell -1}$	𝒰(−1,1)
local weight $\sigma_{L}$	0.03^$^
noise on *B* = *W*^*T*^	𝒰(−0.5,0.5)

^‡^ As we only train deterministic image generation, the train, validation and test sets are all equal.

^$^ We down-scaled local weight noise compared to previous experiments, to avoid exploding activity due to local all-to-all connectivity. In biology, this may be mitigated by a distance rule (see Discussion).

For the simulation parameters for the Yin-Yang task (Fig 3), see [74]. All values were chosen the same, with special parameters $\sigma_{L}=0.1$ and noise on $B=W^{T}$ sampled from 𝒰(−0.1,0.1) for our error microcircuit model.

## Supporting information

S1 Text The supplementary file contains all additional explanations and figures cited in the main text. [122–124] It includes the following sections: Additional simulation results, Implementation of dendritic hierarchical PC, Alternative description of our model with top-down errors in representation dendrites, Re: “Vectorized instructive signals in cortical dendrites during a brain-computer interface task”, Effective functional interareal connectivity during visually guided behavior in mice accomodates our model, Alternative connectivity with inter-area L5 → L2/3 projections. Relaxing the one-to-one matching of representation and error units, Neuronal dynamics before and after learning.
(PDF)

## References

[1] Lillicrap TP, Santoro A, Marris L, Akerman CJ, Hinton G. Backpropagation and the brain. Nat Rev Neurosci. 2020;21(6):335–46. doi: 10.1038/s41583-020-0277-3 32303713

[2] Lillicrap TP, Santoro A. Backpropagation through time and the brain. Curr Opin Neurobiol. 2019;55:82–9. doi: 10.1016/j.conb.2019.01.011 30851654

[3] Crick F. The recent excitement about neural networks. Nature. 1989;337(6203):129–32. doi: 10.1038/337129a0 2911347

[4] Thomas ER, Haarsma J, Nicholson J, Yon D, Kok P, Press C. Predictions and errors are distinctly represented across V1 layers. Curr Biol. 2024;34(10):2265-2271.e4. doi: 10.1016/j.cub.2024.04.036 38697110

[5] Schultz W, Dayan P, Montague PR. A neural substrate of prediction and reward. Science. 1997;275(5306):1593–9. doi: 10.1126/science.275.5306.1593 9054347

[6] Schultz W. Predictive reward signal of dopamine neurons. J Neurophysiol. 1998. doi: 10.1152/jn.1998.80.1.1 9658025

[7] Audette NJ, Schneider DM. Stimulus-Specific Prediction Error Neurons in Mouse Auditory Cortex. J Neurosci. 2023;43(43):7119–29. doi: 10.1523/JNEUROSCI.0512-23.2023 37699716 PMC10601367

[8] Ayaz A, Stäuble A, Hamada M, Wulf M-A, Saleem AB, Helmchen F. Layer-specific integration of locomotion and sensory information in mouse barrel cortex. Nat Commun. 2019;10(1):2585. doi: 10.1038/s41467-019-10564-8 31197148 PMC6565743

[9] Fiser A, Mahringer D, Oyibo HK, Petersen AV, Leinweber M, Keller GB. Experience-dependent spatial expectations in mouse visual cortex. Nat Neurosci. 2016;19(12):1658–64. doi: 10.1038/nn.4385 27618309

[10] Heindorf M, Arber S, Keller GB. Mouse Motor Cortex Coordinates the Behavioral Response to Unpredicted Sensory Feedback. Neuron. 2018;99(5):1040-1054.e5. doi: 10.1016/j.neuron.2018.07.046 30146302 PMC6127035

[11] Keller GB, Bonhoeffer T, Hübener M. Sensorimotor mismatch signals in primary visual cortex of the behaving mouse. Neuron. 2012;74(5):809–15. doi: 10.1016/j.neuron.2012.03.040 22681686

[12] Keller GB, Hahnloser RHR. Neural processing of auditory feedback during vocal practice in a songbird. Nature. 2009;457(7226):187–90. doi: 10.1038/nature07467 19005471

[13] Zmarz P, Keller GB. Mismatch Receptive Fields in Mouse Visual Cortex. Neuron. 2016;92(4):766–72. doi: 10.1016/j.neuron.2016.09.057 27974161

[14] Makino H, Komiyama T. Learning enhances the relative impact of top-down processing in the visual cortex. Nat Neurosci. 2015;18(8):1116–22. doi: 10.1038/nn.4061 26167904 PMC4523093

[15] Jordan R, Keller GB. Opposing Influence of Top-down and Bottom-up Input on Excitatory Layer 2/3 Neurons in Mouse Primary Visual Cortex. Neuron. 2020;108(6):1194-1206.e5. doi: 10.1016/j.neuron.2020.09.024 33091338 PMC7772056

[16] Gillon CJ, Pina JE, Lecoq JA, Ahmed R, Billeh YN, Caldejon S. Learning from unexpected events in the neocortical microcircuit. BioRxiv. 2021. 2021–01.

[17] Attinger A, Wang B, Keller GB. Visuomotor Coupling Shapes the Functional Development of Mouse Visual Cortex. Cell. 2017;169(7):1291-1302.e14. doi: 10.1016/j.cell.2017.05.023 28602353

[18] Ebina T, Masamizu Y, Tanaka YR, Watakabe A, Hirakawa R, Hirayama Y. Two-photon imaging of neuronal activity in motor cortex of marmosets during upper-limb movement tasks. Nature Communications. 2018;9(1):1879. doi: 10.1038/s41467-018-04286-6 PMC5951821 29760466

[19] Obara K, Ebina T, Terada S-I, Uka T, Komatsu M, Takaji M, et al. Change detection in the primate auditory cortex through feedback of prediction error signals. Nat Commun. 2023;14(1):6981. doi: 10.1038/s41467-023-42553-3 37957168 PMC10643402

[20] Ali A, Ahmad N, de Groot E, Johannes van Gerven MA, Kietzmann TC. Predictive coding is a consequence of energy efficiency in recurrent neural networks. Patterns (N Y). 2022;3(12):100639. doi: 10.1016/j.patter.2022.100639 36569556 PMC9768680

[21] Whittington JCR, Bogacz R. An approximation of the error backpropagation algorithm in a predictive coding network with local hebbian synaptic plasticity. Neural Computation. 2017;29(5):1229–62. doi: 10.1162/NECO_a_00949 28333583 PMC5467749

[22] Whittington JCR, Bogacz R. Theories of Error Back-Propagation in the Brain. Trends Cogn Sci. 2019;23(3):235–50. doi: 10.1016/j.tics.2018.12.005 30704969 PMC6382460

[23] Millidge B, Tschantz A, Buckley CL. Predictive Coding Approximates Backprop Along Arbitrary Computation Graphs. Neural Comput. 2022;34(6):1329–68. doi: 10.1162/neco_a_01497 35534010

[24] Song Y, Lukasiewicz T, Xu Z, Bogacz R. Can the Brain Do Backpropagation? -Exact Implementation of Backpropagation in Predictive Coding Networks. Adv Neural Inf Process Syst. 2020;33:22566–79. 33840988 PMC7610561

[25] Mikulasch FA, Rudelt L, Wibral M, Priesemann V. Where is the error? Hierarchical predictive coding through dendritic error computation. Trends Neurosci. 2023;46(1):45–59. doi: 10.1016/j.tins.2022.09.007 36577388

[26] Rosenbaum R. On the relationship between predictive coding and backpropagation. PLoS One. 2022;17(3):e0266102. doi: 10.1371/journal.pone.0266102 35358258 PMC8970408

[27] Pinchetti L, Qi C, Lokshyn O, Olivers G, Emde C, Tang M. Benchmarking Predictive Coding Networks–Made Simple. arXiv preprint. 2024. doi: arXiv:240701163

[28] Innocenti F, Achour EM, Buckley CL. Eqn281 PC: Scaling Predictive Coding to 100 Layer Networks. In: 2025. https://doi.org/arXiv:250513124

[29] Rao RP, Ballard DH. Predictive coding in the visual cortex: a functional interpretation of some extra-classical receptive-field effects. Nat Neurosci. 1999;2(1):79–87. doi: 10.1038/4580 10195184

[30] Sacramento J, Ponte Costa R, Bengio Y, Senn W. Dendritic cortical microcircuits approximate the backpropagation algorithm. Advances in Neural Information Processing Systems. 2018;31.

[31] Cornford JH, Mercier MS, Leite M, Magloire V, Häusser M, Kullmann DM. Dendritic NMDA receptors in parvalbumin neurons enable strong and stable neuronal assemblies. Elife. 2019;8:e49872. doi: 10.7554/eLife.49872 31657720 PMC6839945

[32] Van Ooyen A, Roelfsema P, Kaynak O, Alaydin E, OJa E, Xu L. In: Supplementary Proceedings of the International Conference on Artificial Neural Networks, 2003. 442–4.

[33] Pozzi I, Bohté S, Roelfsema P. A biologically plausible learning rule for deep learning in the brain. arXiv preprint arXiv:181101768. 2018;.

[34] Pozzi I, Bohte S, Roelfsema P. Attention-gated brain propagation: How the brain can implement reward-based error backpropagation. Advances in Neural Information Processing Systems. Curran Associates, Inc. 2020. p. 2516–26.

[35] Lei J, Benjamin AS, Kording KP. Object based attention through internal gating. 2021. https://arxiv.org/abs/2106.04540

[36] Zambrano D, Rombouts J, Laschi C, Bohte S. Spiking AGREL. Signal. 2014;2(3):3–5.

[37] Scellier B, Bengio Y. Equilibrium Propagation: Bridging the Gap Between Energy-Based Models and Backpropagation. 2017. 10.3389/fncom.2017.00024 PMC5415673 28522969

[38] Meulemans A, Tristany Farinha M, Garcia Ordonez J, Vilimelis Aceituno P, Sacramento J, Grewe BF. Credit assignment in neural networks through deep feedback control. Advances in Neural Information Processing Systems. 2021;34.

[39] Greedy W, Zhu HW, Pemberton J, Mellor J, Costa RP. Single-phase deep learning in cortico-cortical networks. 2022. https://arxiv.org/abs/2206.11769

[40] Markov NT, Ercsey-Ravasz MM, Ribeiro Gomes AR, Lamy C, Magrou L, Vezoli J, et al. A weighted and directed interareal connectivity matrix for macaque cerebral cortex. Cereb Cortex. 2014;24(1):17–36. doi: 10.1093/cercor/bhs270 23010748 PMC3862262

[41] Spruston N. Pyramidal neurons: dendritic structure and synaptic integration. Nat Rev Neurosci. 2008;9(3):206–21. doi: 10.1038/nrn2286 18270515

[42] Larkum M. A cellular mechanism for cortical associations: an organizing principle for the cerebral cortex. Trends Neurosci. 2013;36(3):141–51. doi: 10.1016/j.tins.2012.11.006 23273272

[43] Haider P, Ellenberger B, Kriener L, Jordan J, Senn W, Petrovici MA. Latent Equilibrium: A Unified Learning Theory for Arbitrarily Fast Computation with Arbitrarily Slow Neurons. Advances in Neural Information Processing Systems. 2021;34:17839–51.

[44] Ellenberger B, Haider P, Benitez F, Jordan J, Max K, Jaras I, et al. Backpropagation through space, time and the brain. Nat Commun. 2025;17(1):66. doi: 10.1038/s41467-025-66666-z 41453943 PMC12770517

[45] O’Reilly RC. Biologically plausible error-driven learning using local activation differences: The generalized recirculation algorithm. Neural Computation. 1996;8(5):895–938. doi: 10.1162/neco.1996.8.5.895

[46] Xie X, Seung HS. Equivalence of backpropagation and contrastive Hebbian learning in a layered network. Neural Comput. 2003;15(2):441–54. doi: 10.1162/089976603762552988 12590814

[47] Hinton GE, Dayan P, Frey BJ, Neal RM. The “wake-sleep” algorithm for unsupervised neural networks. Science. 1995;268(5214):1158–61. doi: 10.1126/science.7761831 7761831

[48] Lee D-H, Zhang S, Fischer A, Bengio Y. Difference Target Propagation. Lecture Notes in Computer Science. Springer International Publishing. 2015. p. 498–515. 10.1007/978-3-319-23528-8_31

[49] Meulemans A, Carzaniga F, Suykens J, Sacramento J, Grewe BF. A theoretical framework for target propagation. Advances in Neural Information Processing Systems. 2020;33:20024–36.

[50] Ernoult MM, Normandin F, Moudgil A, Spinney S, Belilovsky E, Rish I. In: International Conference on Machine Learning, 2022. 5968–87.

[51] Roelfsema P, Ooyen A. Attention-gated reinforcement learning of internal representations for classification. Cogn Sci. 17:2176–214. doi: 10.1162/0899766054615699 16105222

[52] Markov NT, Vezoli J, Chameau P, Falchier A, Quilodran R, Huissoud C, et al. Anatomy of hierarchy: feedforward and feedback pathways in macaque visual cortex. J Comp Neurol. 2014;522(1):225–59. doi: 10.1002/cne.23458 23983048 PMC4255240

[53] Siegle JH, Jia X, Durand S, Gale S, Bennett C, Graddis N, et al. Survey of spiking in the mouse visual system reveals functional hierarchy. Nature. 2021;592(7852):86–92. doi: 10.1038/s41586-020-03171-x 33473216 PMC10399640

[54] Peng Y, Bjelde A, Aceituno PV, Mittermaier FX, Planert H, Grosser S, et al. Directed and acyclic synaptic connectivity in the human layer 2-3 cortical microcircuit. Science. 2024;384(6693):338–43. doi: 10.1126/science.adg8828 38635709

[55] Petreanu L, Mao T, Sternson SM, Svoboda K. The subcellular organization of neocortical excitatory connections. Nature. 2009;457(7233):1142–5. doi: 10.1038/nature07709 19151697 PMC2745650

[56] Francioni V, Tang VD, Brown NJ, Toloza EHS, Harnett M. Vectorized instructive signals in cortical dendrites during a brain-computer interface task. 2023. doi: 10.1101/2023.11.03.565534 PMC13112360 41741650

[57] Funamizu A, Kuhn B, Doya K. Neural substrate of dynamic Bayesian inference in the cerebral cortex. Nat Neurosci. 2016;19(12):1682–9. doi: 10.1038/nn.4390 27643432

[58] Larkum ME, Senn W, Lüscher H-R. Top-down dendritic input increases the gain of layer 5 pyramidal neurons. Cereb Cortex. 2004;14(10):1059–70. doi: 10.1093/cercor/bhh065 15115747

[59] Quiquempoix M, Fayad SL, Boutourlinsky K, Leresche N, Lambert RC, Bessaih T. Layer 2/3 Pyramidal Neurons Control the Gain of Cortical Output. Cell Rep. 2018;24(11):2799-2807.e4. doi: 10.1016/j.celrep.2018.08.038 30208307

[60] Aceituno PV, Farinha MT, Loidl R, Grewe BF. Learning cortical hierarchies with temporal Hebbian updates. 2023. 10.3389/fncom.2023.1136010 PMC10244748 37293353

[61] Plesser HE, Gerstner W. Escape rate models for noisy integrate-and-free neurons. Neurocomputing. 2000;32–33:219–24. doi: 10.1016/s0925-2312(00)00167-3

[62] Köndgen H, Geisler C, Fusi S, Wang X-J, Lüscher H-R, Giugliano M. The dynamical response properties of neocortical neurons to temporally modulated noisy inputs in vitro. Cereb Cortex. 2008;18(9):2086–97. doi: 10.1093/cercor/bhm235 18263893 PMC3140196

[63] Brandt S, Petrovici MA, Senn W, Wilmes KA, Benitez F. Prospective and Retrospective Coding in Cortical Neurons. 2024.

[64] Ferguson KA, Cardin JA. Mechanisms underlying gain modulation in the cortex. Nat Rev Neurosci. 2020;21(2):80–92. doi: 10.1038/s41583-019-0253-y 31911627 PMC7408409

[65] Keller GB, Mrsic-Flogel TD. Predictive Processing: A Canonical Cortical Computation. Neuron. 2018;100(2):424–35. doi: 10.1016/j.neuron.2018.10.003 30359606 PMC6400266

[66] Wilmes KA, Petrovici MA, Sachidhanandam S, Senn W. Uncertainty-modulated prediction errors in cortical microcircuits. Elife. 2025;13:RP95127. doi: 10.7554/eLife.95127 40471208 PMC12140629

[67] Spratling MW. Reconciling predictive coding and biased competition models of cortical function. Front Comput Neurosci. 2008;2:4. doi: 10.3389/neuro.10.004.2008 18978957 PMC2576514

[68] Spratling MW. Predictive coding as a model of biased competition in visual attention. Vision Res. 2008;48(12):1391–408. doi: 10.1016/j.visres.2008.03.009 18442841

[69] Spratling MW. Fitting predictive coding to the neurophysiological data. Brain Res. 2019;1720:146313. doi: 10.1016/j.brainres.2019.146313 31265817

[70] Hertäg L, Sprekeler H. Learning prediction error neurons in a canonical interneuron circuit. Elife. 2020;9:e57541. doi: 10.7554/eLife.57541 32820723 PMC7442488

[71] Urbanczik R, Senn W. Learning by the dendritic prediction of somatic spiking. Neuron. 2014;81(3):521–8. doi: 10.1016/j.neuron.2013.11.030 24507189

[72] Kolen JF, Pollack JB. Backpropagation without weight transport. In: Proceedings of 1994 IEEE International Conference on Neural Networks (ICNN’94). 1375–80. 10.1109/icnn.1994.374486

[73] Lillicrap TP, Cownden D, Tweed DB, Akerman CJ. Random synaptic feedback weights support error backpropagation for deep learning. Nat Commun. 2016;7:13276. doi: 10.1038/ncomms13276 27824044 PMC5105169

[74] Max K, Kriener L, Pineda García G, Nowotny T, Jaras I, Senn W, et al. Learning efficient backprojections across cortical hierarchies in real time. Nat Mach Intell. 2024;6(6):619–30. doi: 10.1038/s42256-024-00845-3

[75] Rungratsameetaweemana N, Kim R, Chotibut T, Sejnowski TJ. Random noise promotes slow heterogeneous synaptic dynamics important for robust working memory computation. Proc Natl Acad Sci U S A. 2025;122(3):e2316745122. doi: 10.1073/pnas.2316745122 39819216 PMC11760912

[76] Kriener L, Göltz J, Petrovici MA. The Yin-Yang dataset. In: Neuro-Inspired Computational Elements Conference, 2022. 107–11. 10.1145/3517343.3517380

[77] Liao Q, Leibo J, Poggio T. How important is weight symmetry in backpropagation?. AAAI. 2016;30(1). doi: 10.1609/aaai.v30i1.10279

[78] Gierlich T, Baumbach A, Kungl AF, Max K, Petrovici MA. Weight transport through spike timing for robust local gradients. arXiv. 2025. doi: 10.48550/arXiv.2503.02642

[79] Marschall O, Cho K, Savin C. A unified framework of online learning algorithms for training recurrent neural networks. Journal of Machine Learning Research. 2020;21(135):1–34.34305477

[80] Walsh KS, McGovern DP, Clark A, O’Connell RG. Evaluating the neurophysiological evidence for predictive processing as a model of perception. Ann N Y Acad Sci. 2020;1464(1):242–68. doi: 10.1111/nyas.14321 32147856 PMC7187369

[81] Rossbroich J, Zenke F. Dis-inhibitory neuronal circuits can control the sign of synaptic plasticity. In: Advances in Neural Information Processing Systems 36, 2023. 64059–82. 10.52202/075280-2799

[82] Bastos AM, Usrey WM, Adams RA, Mangun GR, Fries P, Friston KJ. Canonical microcircuits for predictive coding. Neuron. 2012;76(4):695–711. doi: 10.1016/j.neuron.2012.10.038 23177956 PMC3777738

[83] Aceituno PV, de Haan S, Loidl R, Grewe BF. Challenging Backpropagation: Evidence for Target Learning in the Cortex. bioRxiv. 2024.

[84] Silver RA. Neuronal arithmetic. Nat Rev Neurosci. 2010;11(7):474–89. doi: 10.1038/nrn2864 20531421 PMC4750293

[85] Staiger JF, Petersen CCH. Neuronal Circuits in Barrel Cortex for Whisker Sensory Perception. Physiol Rev. 2021;101(1):353–415. doi: 10.1152/physrev.00019.2019 32816652

[86] Bureau I, von Saint Paul F, Svoboda K. Interdigitated paralemniscal and lemniscal pathways in the mouse barrel cortex. PLoS Biol. 2006;4(12):e382. doi: 10.1371/journal.pbio.0040382 17121453 PMC1637129

[87] Staiger JF, Bojak I, Miceli S, Schubert D. A gradual depth-dependent change in connectivity features of supragranular pyramidal cells in rat barrel cortex. Brain Struct Funct. 2015;220(3):1317–37. doi: 10.1007/s00429-014-0726-8 24569853 PMC4409644

[88] Hage TA, Bosma-Moody A, Baker CA, Kratz MB, Campagnola L, Jarsky T, et al. Synaptic connectivity to L2/3 of primary visual cortex measured by two-photon optogenetic stimulation. Elife. 2022;11:e71103. doi: 10.7554/eLife.71103 35060903 PMC8824465

[89] Rossa M. Interlaminar connectivity in mouse primary visual cortex. UC San Diego. 2023.

[90] Lefort S, Tomm C, Floyd Sarria J-C, Petersen CCH. The excitatory neuronal network of the C2 barrel column in mouse primary somatosensory cortex. Neuron. 2009;61(2):301–16. doi: 10.1016/j.neuron.2008.12.020 19186171

[91] Douglas RJ, Martin KAC. Neuronal circuits of the neocortex. Annu Rev Neurosci. 2004;27:419–51. doi: 10.1146/annurev.neuro.27.070203.144152 15217339

[92] Harris KD, Shepherd GMG. The neocortical circuit: themes and variations. Nat Neurosci. 2015;18(2):170–81. doi: 10.1038/nn.3917 25622573 PMC4889215

[93] Ercsey-Ravasz M, Markov NT, Lamy C, Van Essen DC, Knoblauch K, Toroczkai Z, et al. A predictive network model of cerebral cortical connectivity based on a distance rule. Neuron. 2013;80(1):184–97. doi: 10.1016/j.neuron.2013.07.036 24094111 PMC3954498

[94] Gămănuţ R, Kennedy H, Toroczkai Z, Ercsey-Ravasz M, Van Essen DC, Knoblauch K, et al. The Mouse Cortical Connectome, Characterized by an Ultra-Dense Cortical Graph, Maintains Specificity by Distinct Connectivity Profiles. Neuron. 2018;97(3):698-715.e10. doi: 10.1016/j.neuron.2017.12.037 29420935 PMC5958229

[95] Horvát S, Gămănuț R, Ercsey-Ravasz M, Magrou L, Gămănuț B, Van Essen DC, et al. Spatial Embedding and Wiring Cost Constrain the Functional Layout of the Cortical Network of Rodents and Primates. PLoS Biol. 2016;14(7):e1002512. doi: 10.1371/journal.pbio.1002512 27441598 PMC4956175

[96] Moskovitz TH, Litwin-Kumar A, Abbott L. Feedback alignment in deep convolutional networks. arXiv preprint. 2018. https://arxiv.org/abs/1812.06488

[97] Kuo Y-C. ML14: Pytorch - MLP on MNIST. In: 2021.

[98] Zhu Y. The Drosophila visual system: From neural circuits to behavior. Cell Adh Migr. 2013;7(4):333–44. doi: 10.4161/cam.25521 23880926 PMC3739809

[99] D’Souza RD, Wang Q, Ji W, Meier AM, Kennedy H, Knoblauch K, et al. Hierarchical and nonhierarchical features of the mouse visual cortical network. Nat Commun. 2022;13(1):503. doi: 10.1038/s41467-022-28035-y 35082302 PMC8791996

[100] Burkhalter A, D’Souza RD, Ji W, Meier AM. Integration of Feedforward and Feedback Information Streams in the Modular Architecture of Mouse Visual Cortex. Annu Rev Neurosci. 2023;46:259–80. doi: 10.1146/annurev-neuro-083122-021241 36972612

[101] Winding M, Pedigo BD, Barnes CL, Patsolic HG, Park Y, Kazimiers T, et al. The connectome of an insect brain. Science. 2023;379(6636):eadd9330. doi: 10.1126/science.add9330 36893230 PMC7614541

[102] López FM, Triesch J. Hierarchical Residuals Exploit Brain-Inspired Compositionality. In: 2025. https://doi.org/arXiv:250216003

[103] Williams RJ, Zipser D. Gradient-based learning algorithms for recurrent connectionist networks. In: 1990.

[104] Murray JM. Local online learning in recurrent networks with random feedback. Elife. 2019;8:e43299. doi: 10.7554/eLife.43299 31124785 PMC6561704

[105] Senn W, Dold D, Kungl AF, Ellenberger B, Jordan J, Bengio Y, et al. A neuronal least-action principle for real-time learning in cortical circuits. Elife. 2024;12:RP89674. doi: 10.7554/eLife.89674 39704647 PMC11661794

[106] Meulemans A, Farinha MT, Cervera MR, Sacramento J, Grewe BF. Minimizing control for credit assignment with strong feedback. 2022.

[107] Laborieux A, Zenke F. Holomorphic Equilibrium Propagation Computes Exact Gradients Through Finite Size Oscillations. In: Advances in Neural Information Processing Systems 35, 2022. 12950–63. 10.52202/068431-0941

[108] Balwani AH, Wang AQ, Najafi F, Choi H. Constructing biologically constrained RNNs via Dale’s backprop and topologically-informed pruning. bioRxiv. 2025;:2025.01.09.632231. 10.1126/sciadv.adw4970 PMC12700192 41385638

[109] Cornford J, Kalajdzievski D, Leite M, Lamarquette A, Kullmann DM, Richards B. Learning to live with Dale’s principle: ANNs with separate excitatory and inhibitory units. In: bioRxiv and ICLR 2021 Poster, 2021. 10.1101/2020.11.02.364968

[110] Sermet BS, Truschow P, Feyerabend M, Mayrhofer JM, Oram TB, Yizhar O, et al. Pathway-, layer- and cell-type-specific thalamic input to mouse barrel cortex. Elife. 2019;8:e52665. doi: 10.7554/eLife.52665 31860443 PMC6924959

[111] Munn BR, Müller EJ, Aru J, Whyte CJ, Gidon A, Larkum ME, et al. A thalamocortical substrate for integrated information via critical synchronous bursting. Proc Natl Acad Sci U S A. 2023;120(46):e2308670120. doi: 10.1073/pnas.2308670120 37939085 PMC10655573

[112] Suzuki M, Pennartz CMA, Aru J. How deep is the brain? The shallow brain hypothesis. Nat Rev Neurosci. 2023;24(12):778–91. doi: 10.1038/s41583-023-00756-z 37891398

[113] Furutachi S, Franklin AD, Aldea AM, Mrsic-Flogel TD, Hofer SB. Cooperative thalamocortical circuit mechanism for sensory prediction errors. Nature. 2024;633(8029):398–406. doi: 10.1038/s41586-024-07851-w 39198646 PMC11390482

[114] Sherman SM, Usrey WM. Transthalamic Pathways for Cortical Function. J Neurosci. 2024;44(35):e0909242024. doi: 10.1523/JNEUROSCI.0909-24.2024 39197951 PMC11358609

[115] Mo C, McKinnon C, Murray Sherman S. A transthalamic pathway crucial for perception. Nat Commun. 2024;15(1):6300. doi: 10.1038/s41467-024-50163-w 39060240 PMC11282105

[116] McKinnon C, Mo C, Sherman SM. Disruption of transthalamic circuitry from primary visual cortex impairs visual discrimination in mice. J Neurosci. 2025;45(18):e0002252025. doi: 10.1523/JNEUROSCI.0002-25.2025 40139804 PMC12044039

[117] Yamins DLK, Hong H, Cadieu CF, Solomon EA, Seibert D, DiCarlo JJ. Performance-optimized hierarchical models predict neural responses in higher visual cortex. Proc Natl Acad Sci U S A. 2014;111(23):8619–24. doi: 10.1073/pnas.1403112111 24812127 PMC4060707

[118] Nøkland A. Direct feedback alignment provides learning in deep neural networks. Advances in Neural Information Processing Systems. 2016;29.

[119] Bartunov S, Santoro A, Richards B, Marris L, Hinton GE, Lillicrap T. Assessing the scalability of biologically-motivated deep learning algorithms and architectures. Advances in Neural Information Processing Systems. 2018;31.

[120] Lansdell BJ, Prakash PR, Kording KP. Learning to solve the credit assignment problem. arXiv preprint. 2019. doi: 10.48550/arXiv.1906.00889

[121] Millidge B, Seth A, Buckley CL. Predictive coding: a theoretical and experimental review. arXiv preprint. 2021. doi: arXiv:2107.12979

[123] Haeusler S, Maass W. A statistical analysis of information-processing properties of lamina-specific cortical microcircuit models. Cereb Cortex. 2007;17(1):149–62. doi: 10.1093/cercor/bhj132 16481565

[124] Shipp S. The functional logic of corticostriatal connections. Brain Struct Funct. 2017;222(2):669–706. doi: 10.1007/s00429-016-1250-9 27412682 PMC5334428

[125] Wang L, Nour Eddine S, Brothers T, Jensen O, Kuperberg GR. An implemented predictive coding model of lexico-semantic processing explains the dynamics of univariate and multivariate activity within the left ventromedial temporal lobe during reading comprehension. Neuroimage. 2025;308:120977. doi: 10.1016/j.neuroimage.2024.120977 39694345 PMC11894502

