# Supplementary material for: ‘Backpropagation and the brain’ realized in cortical error neuron microcircuits
Source: PLoS Comput Biol. 2026 Apr 17;22(4):e1014164. doi: 10.1371/journal.pcbi.1014164 (PMC13089762; doi:10.1371/journal.pcbi.1014164)
Supplement: S1 Text — It includes the following sections: Additional simulation results, Implementation of dendritic hierarchical PC, Alternative description of our model with top-down errors in representation dendrites, Re: “Vectorized instructive signals in cortical dendrites during a brain-computer interface task”, Effective functional interareal connectivity during visually guided behavior in mice accomodates our model, Alternative connectivity with inter-area L5 → L2/3 projections. Relaxing the one-to-one matching of representation and error units, Neuronal dynamics before and after learning. (PDF) [file pcbi.1014164.s001.pdf]

S1 Text

S1.1. Additional simulation results

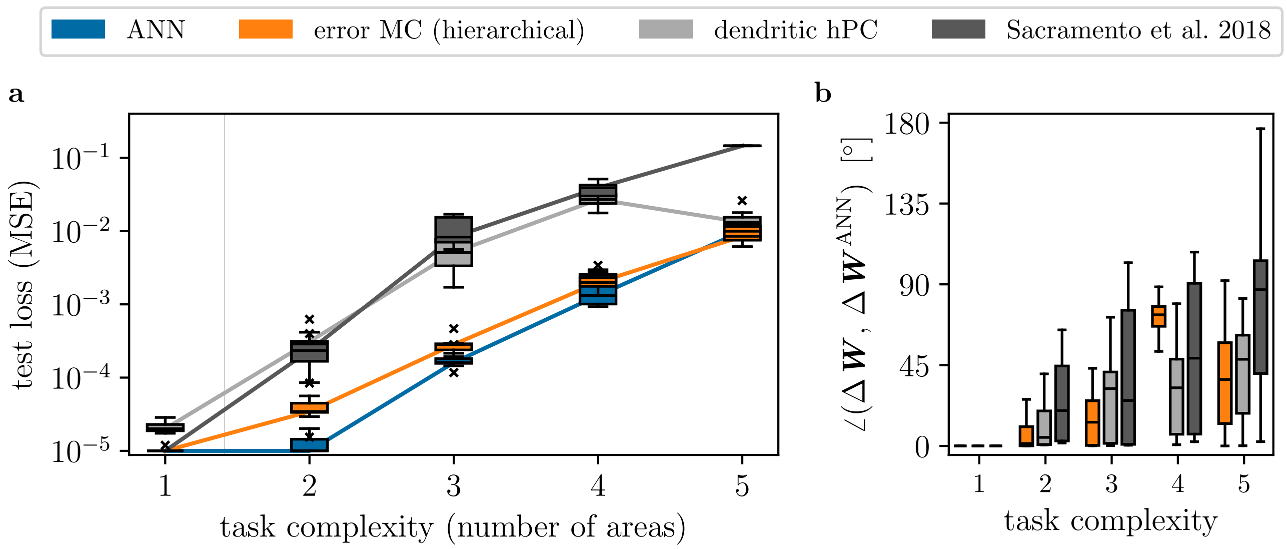

Fig A: error neuron microcircuit performs equally well with realistic *and* strictly hierarchical connectivity. As opposed to dendritic hPC and Sacramento et al. [30], our model implements skip connectivity. To demonstrate that the difference in performance does not stem from the different architectures, we repeat the experiment of Fig 4 with strictly hierarchical connectivity for the error neuron microcircuits. Note that performance is essentially unaffected, while the weight updates are much closer to those of an ANN.

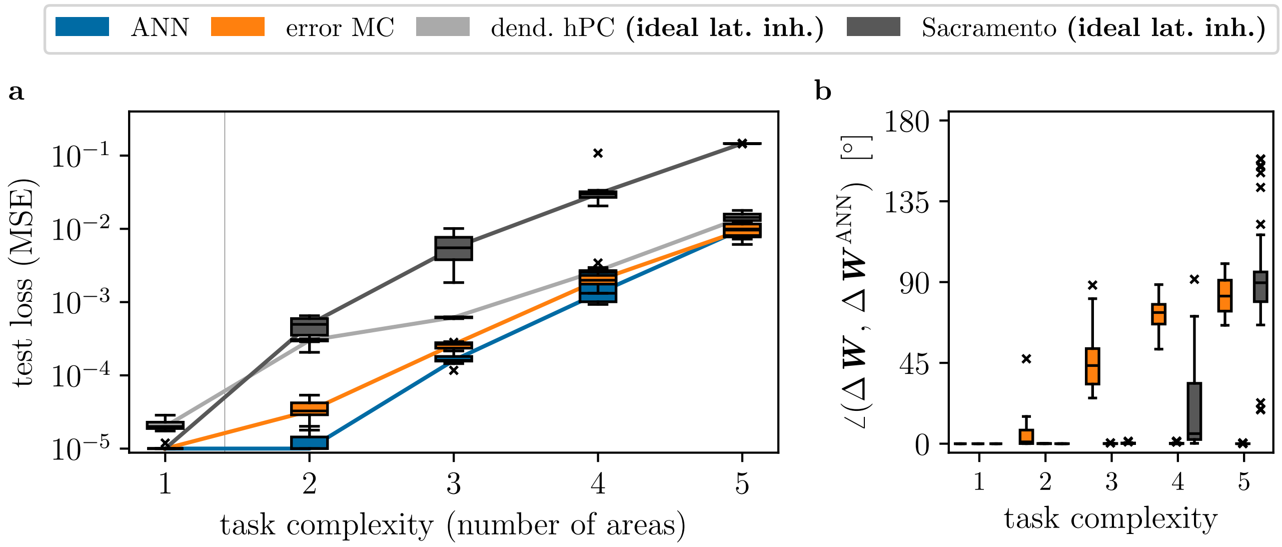

Fig B: Dendritic error construction requires ideal lateral inhibition.
Models where errors are constructed on dendrites (instead of explicit error neurons) require tight balance between top-down prediction and lateral inhibition. In Fig 4, we have shown that such models do not scale to many areas. We argue that one of the reasons is learning of imperfect lateral weights when training multiple areas. To demonstrate this, we repeat the experiment with lateral inhibition set to ideal weights during learning. We observe that dendritic hPC performs much better under such artificial weight copying, but not on par with error neuron microcircuits (without weight transfer) or the ANN.

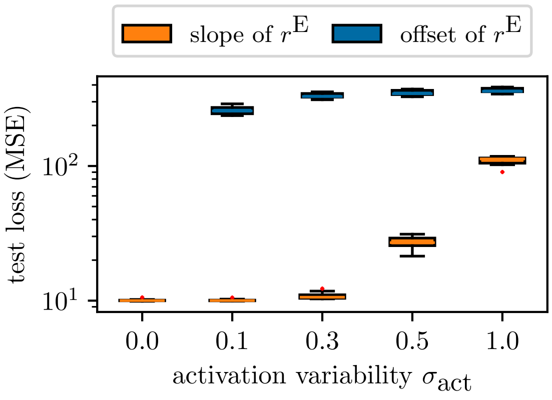

Fig C: Network performance is highly sensitive to offsets of error signals.
We repeat the experiment of Fig 6 a, simulating variability only on the activation functions of error neurons, either on the slope or offset. Under slope variability, performance is stable, with similar results to offset and slope variability on representation units (Fig 6 a). However, any variability on the offset immediately hinders learning, demonstrating the dependence of the network on useful error signals.

S1.2. Implementation of dendritic hierarchical PC

We have adapted the model by Mikulasch et al. [25] in the following way. As explained in Figure 2 of [25], dendritic hPC can be related to the model of Sacramento et al. [30] by inverting the hierarchy of cortical areas. This amounts to a generative and classifying configuration, respectively (see Fig 1). As we are comparing the networks on an arbitrary, deterministic input-output mapping task (cf. Fig 4), there is no actual distinction between generative and classifying configurations; i.e., labels and targets are defined arbitrarily and can be exchanged. To allow for a fair comparison across all models, we therefore swap input and target for dendritic hPC. This effectively trains the network to solve the same task as all other models, which are run in their classification configuration. For example, for 3 areas, all networks receive 8 inputs and process them using three areas with $[4, 2, 1]$ neurons.

We implement dendritic hPC following equations VIII to XIII in Box 2 of [25]. By exchanging basal and apical compartment labels and rewriting the soma dynamics, one can see that dendritic hPC is in fact largely computationally equivalent to the model by Sacramento et al.: Starting from the dendritic microcircuit model [30], we can set $u_k^{\mathrm{I}} = r_k^{\mathrm{P}}$, drop the weight $W_{k,k}^{\mathrm{IP}}$ from the theory, and equate the swapped compartments, $v_{\mathrm{B}}^{\mathrm{P}} \leftrightarrow a$ and $v_{\mathrm{A}}^{\mathrm{P}} \leftrightarrow b$. Soma and dendrite dynamics can be seen to be equivalent by redefining $v_{\mathrm{B,k}}^{\mathrm{P}} = W_{k,k-1}^{\mathrm{PP}} r_{k-1}^{\mathrm{P}} \mapsto v_{\mathrm{B,k}}^{\mathrm{P}} = W_{k,k-1}^{\mathrm{PP}} r_{k-1}^{\mathrm{P}} - r_k^{\mathrm{P}}$. Conductances can be chosen to mirror the dynamics of Eqs. VIII, IX and XI. Weights are then related by $W_{k,k-1}^{\mathrm{PP}} \leftrightarrow D^{\mathrm{i+1}}$ (Eq. XIII), $W_{k,k+1}^{\mathrm{PP}} \leftrightarrow D^{\mathrm{i}}$ (Eq. XII), and $W_{k,k}^{\mathrm{PI}} \leftrightarrow W^{\mathrm{i}}$ (Eq. X).

To establish a fair comparison, we further make the following changes:

- we set both compartments to be instantaneous, $\tau_{\mathrm{b}} = \tau_{\mathrm{a}} = 0$, equal to the other models in this work.
- we implement the same approximation of PAL as in the other models by setting $D_{\mathrm{kj}}^{\mathrm{i}} = [D_{\mathrm{jk}}^{\mathrm{i}}]^{\mathrm{T}} + \Xi$, with Ξ fixed noise. This means that we do not implement Eq. XII. It also greatly reduces computational complexity, as the index k in $W_{\mathrm{jkl}}^{\mathrm{i}}$ can be dropped, reducing the tensor to a matrix.

- the theory of [25] is purely linear. To introduce non-linear activations φ, we re-label the variable r (Eq. XI) to u, representing the soma, and r now representing the output rate.
- to mitigate the relaxation problem, we augment neuron outputs with the prospective coding mechanism, $r = \varphi(u + \tau_r \dot{u})$, as in the other models.
- to account for the non-linear activation functions, we optionally implement the Urbanczik-Senn learning rule in the form $\dot{D}^{i+1}_{jl} = -[r^i_j - \varphi\big(\sum_l D^{i+1}_{jl} r^{i+1}_l\big)]\, r^{i+1}_l$.
- we use the same conductances $\{g_l, g^{rep}, g^{err}\}$ as in the other models (for the model of Sacramento et al., $g^{rep} = g^{bas}$ and $g^{err} = g^{api}$). This can be interpreted as a generalized parametrization of the precision weighting ($\frac{1}{\sigma^2_{i-1}}$ and $\frac{1}{\sigma^2_i}$ in Eq. XI of [25]) of both compartments.

To facilitate tight balance, we set the learning rate of W^i_{jkl} (self-inhibition in area i) to be double that of D^i_{kl} (efferent weights of area i, projecting to areas $i-1$ and $i+1$). Furthermore, we initialize each network with ideal lateral weights, $W^i_{jkl} = D^i_{kj} D^i_{kl}$. We compare the different implementations of dendritic hPC in Fig D.

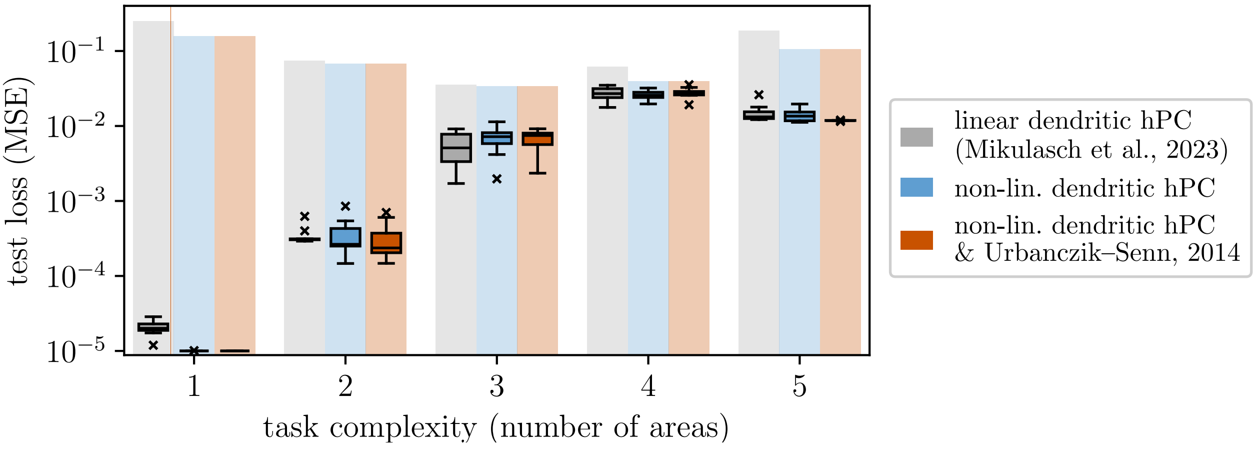

Fig D: Three different implementations of dendritic hPC for learning non-linear tasks. The original model of dendritic hPC by Mikulasch et al. [25] is a purely linear theory. For a fair comparison to our model on a non-trivial benchmark, we implement the original model (grey) and two variants, where linear activations are replaced by non-linearities (light blue), and additionally, the learning rule for D^{i+1}_{jl} is adapted to the Urbanczik-Senn rule [71] (brown). As both modifications do not significantly change performance, we have used the original (grey) model in Fig 4.

S1.3. Alternative description of our model with top-down errors in representation dendrites

As discussed in Section 4, and demonstrated particularly in Fig G, our model admits an alternative parametrization, where representation dendrites encode top-down errors instead of predictions. Here, we show the mathematical equivalence of both parametrizations.

To reformulate our neuron dynamics as in dendritic hPC (Box 2 in [25]), we need to modify the somatic and representation dendrite dynamics of representation units:

$$C_{\mathrm{m}}\dot{u}_\ell^{\mathrm{som}} = \cancel{-g_{\mathrm{l}}u_\ell^{\mathrm{som}}} - g^{\mathrm{rep}}(\cancel{u_\ell^{\mathrm{som}}} - u_\ell^{\mathrm{pred}}) - g^{\mathrm{err}}(\cancel{u_\ell^{\mathrm{som}}} - u_\ell^{\mathrm{err}}) = g^{\mathrm{rep}}u_\ell^{\mathrm{pred}} + g^{\mathrm{err}}u_\ell^{\mathrm{err}} \,, \tag{21}$$

and adding a term to u_ℓ^{pred},

$$u_\ell^{\mathrm{pred}} = \sum_k W_{\ell,k}\, r_k^{\mathrm{R}} - \frac{g_{\mathrm{l}} + g^{\mathrm{rep}} + g^{\mathrm{err}}}{g^{\mathrm{rep}}} u_\ell^{\mathrm{som}} \qquad u_\ell^{\mathrm{err}} = L_{\ell,\ell}^{\mathrm{RE}} r_\ell^{\mathrm{E}} \,. \tag{22}$$

I.e., the representation-receiving compartment now compares the conductance-weighted somatic voltage to top-down predictions. By plugging Equation (22) into Equation (21), one can see that $\dot{u}_\ell^{\mathrm{som}}$ remains unchanged.

We adapt the learning rule of Equation (4) by using the top-down prediction in place of u_ℓ^{pred},

$$\dot{W}_{\ell,m} = \left[\breve{u}_\ell^{\mathrm{som}} - \frac{g^{\mathrm{rep}}}{g_{\mathrm{l}} + g^{\mathrm{rep}} + g^{\mathrm{err}}} \sum_k W_{\ell,k}\, r_k^{\mathrm{R}}\right] r_m^{\mathrm{R}} \,. \tag{23}$$

S1.4. Re: "Vectorized instructive signals in cortical dendrites during a brain-computer interface task"

A recent study of Francioni et al. [56] has analyzed the 'backpropagation and the brain' scenario using optogenetic measurements of L5 PYR in mice performing a brain-computer interface (BCI) task. The authors assign two randomly chosen populations $\mathrm{P}^+/\mathrm{P}^-$, depending on their contribution to solving the task (rotating a bar from 0 to 90 degrees by matching a neuronal target activity). Indeed, they find that the difference between somatic and distal (!) apical

activity correlates with mismatch signals, where the two populations integrate positive and negative errors respectively. The methods applied in this work are highly innovative and pave the way for quantitative studies of error learning on the neuronal level, however several questions remain to be clarified. In the study, the target is defined as a specific difference in activity $P^+ - P^-$. Here, the BCI setup may lead to a confounding of learning and co-activated neurons, as the P^+/P^- populations may include task-unrelated neurons. Furthermore, the task is highly stereotypical, as angle initialization and target are always defined as exactly 0 and 90 degrees respectively, and the target is only approachable from one direction (target $>$ angle always, i.e. task is asymmetric). Thus, the setup is susceptible to habituation, and does not probe all possible error signals (target $>$ angle vs. target $<$ angle); under the 'vector hypothesis' (i.e., backpropagation), one expects to see a sign flip of the error for both populations when changing the direction of approach. It remains to be studied whether their findings extend to more general task setups.

S1.5. Effective functional interareal connectivity during visually guided behavior in mice accomodates our model

A recent analysis by Balwani et al. [106] of visual behavior in mice shows functional connectivity accommodating our circuit and the Rao-Ballard model to varying degrees (see Fig. 5A therein): novel images induce strong feed-forward projections from L2/3 $\rightarrow$ L4 (V1 to LM), which may be passed locally to L2/3 of LM, in agreement with strong same-area projections from L4 to L2/3 in the canonical cortical microcircuit [78, 87, 88, 123, 124]. Such effective functional connectivity may represent our L2/3 $\rightarrow$ L2/3 error stream.

In the analysis of [106], the feedback stream (LM to V1) of L5 neurons shows projections both to L5 but also L2/3; in this direction, it appears that neither our and nor the Rao-Ballard connectivity are cleanly observed, but rather a mixture of both. In our model, this may be explained by L5 representation units mixing predictions and errors (non-weak nudging, 'target-learning'), confounding the distinction of functional projections of error and representation pathways.

S1.6. Alternative connectivity with inter-area L5 $\rightarrow$ L2/3 projections

In [106], Balwani et al. find the feedback stream (LM to V1) to be formed by L5 neurons projection to both L5 and L2/3 across areas. This does not align cleanly with our model, where L5 $\rightarrow$ L2/3 *locally*, nor the classical Rao-Ballard model, where L5 $\rightarrow$ L2/3 in upstream areas, but not L5 $\rightarrow$ L5. This can be accommodated through a slight modification of our model, with an alternative connectivity where L5 representation units do not project locally to error units in the same area, but to error units in upstream areas; see Fig E. Weights L^{ER} now connect representation to error units across areas.

Computationally, the model is slightly modified, affecting error neuron dynamics and the learning rule. Error neurons then compute the backprojected error using the downstream φ', instead of the φ' of the same area. Instead of Equation (12), the compartment voltages are then

$$e_\ell^{\mathrm{rep}} \odot e_\ell^{\mathrm{err}} = [L_{\ell,m}^{\mathrm{ER}} \, \varphi'(\breve{u}_m^{\mathrm{som}})] \odot \sum_m B_{\ell,m} \, r_m^{\mathrm{E}} \, . \tag{24}$$

Because local error signals are now computed without the φ' of a given area, we also modify the learning rule for W of Equation (16),

$$\frac{\mathrm{d}}{\mathrm{dt}} W_{\ell,k} = [\varphi(\breve{u}_\ell^{\mathrm{som}}) - \varphi(\frac{g^{\mathrm{rep}}}{g_\mathrm{l} + g^{\mathrm{rep}} + g^{\mathrm{err}}} u_\ell^{\mathrm{pred}})] \times r_k^{\mathrm{R}} \, , \tag{25}$$

turning it into an Urbanczik-Senn learning rule. For weak nudging, $g^{\mathrm{err}} \ll g_\mathrm{l} + g^{\mathrm{rep}}$, this approximates Equation (16) with an additional factor $\varphi'(\breve{u}_\ell^{\mathrm{som}})$.

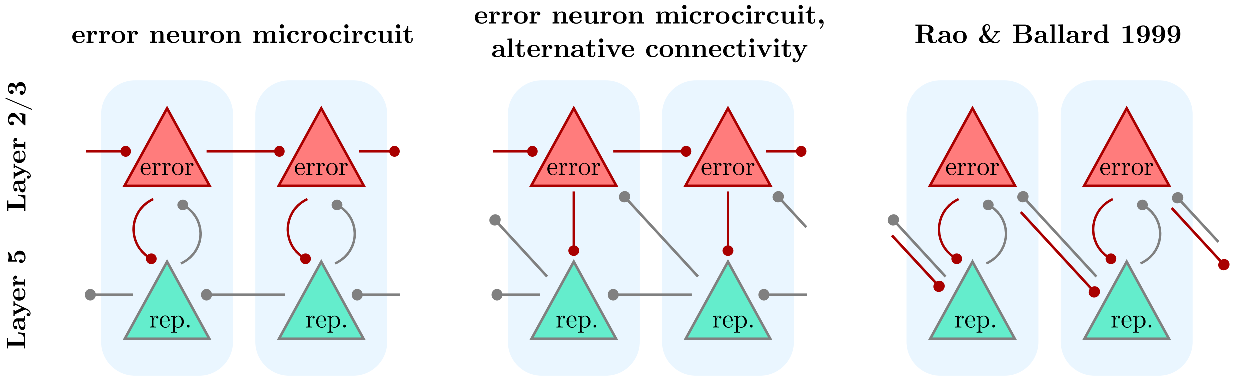

Fig E: Alternative connectivity for our model.

S1.7. Relaxing the one-to-one matching of representation and error units

As opposed to standard implementations of PC, we relax the condition of exact matching of representation and error units. Error units project locally to representation units with $L^{\text{RE}} \sim \mathbf{1} + \mathcal{U}(-\sigma_L, \sigma_L)$, i.e. an underlying one-to-one matching is relaxed by additional, random all-to-all connectivity.

Based on these two terms making up L^{RE}, we can separate a given error neuron population into 'regular' errors, with one-to-one matching, and additional ('excess') error units that broadcast their error signals to the representation units via all-to-all connectivity.

For $n_E > n_R$, excess error units essentially do not affect the network dynamics. For example, for $n_R = 1$ and $n_E = 2$, we can write $L^{\text{RE}} = (1, 0) + (\varepsilon, \varepsilon')$, with $\varepsilon, \varepsilon' \sim \mathcal{U}(-\sigma_L, \sigma_L)$; only the first error neuron is strongly connected to the representation unit, see Fig F a. As $\epsilon' \ll 1$, the excess error only mildly affects the single representation unit, and in practice does not affect performance (see top-left entries in Fig 5 b).

For $n_R > n_E$, the situation is different: as only n_E errors are provided to the representation units (Fig F b), also only n_E representation units perform error-based learning. The remaining $(n_R - n_E)$ representation neurons essentially do not learn (again, as $\epsilon' \ll 1$).

We can understand why their presence can nevertheless improve performance by comparing this setup with ANNs and support vector machines (SVM), see Fig F c. SVMs project their input into a (usually high dimensional) feature space, and train the mapping from that space to the output. Therefore, SVMs can loosely be seen as special ANNs without error propagation

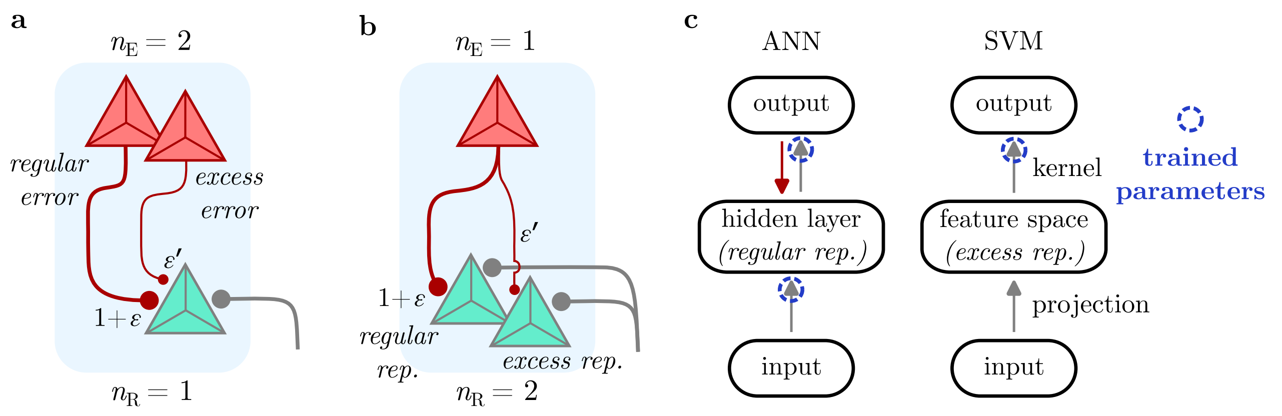

Fig F: Error microcircuits are only weakly affected by additional error units, but benefit from excess representation units. a) Connectivity of an area with two error and one representation units. Preferential targeting implies that there is 'partner error neuron' strongly connected to the representation unit (left error neuron), while the other error neuron has a weaker connection. Excess errors are scaled down, such that learning performance is unaffected. **b)** If there are more representation neurons than error units, the excess representation unit does effectively not learn, but still increases the dimensionality, and thus capacity of a given area to encode representations. **c)** SVMs can loosely be interpreted as two-layer ANNs with only one learnable 'weight'. Representation units which (do not) receive an error are analogous to hidden units (feature vectors) in an ANN (SVM).

and learning in the hidden layer.

We can relate these two setups to the representation neurons in our model: Per construction, representation units which receive an error signal (*regular rep. units*) behave similar to units in an ANN. On the other hand, representation units which do not receive an error signal (*excess rep. units*) are analogous to vectors in the feature space of an SVM. Therefore, for $n_R > n_E$, an area in our model performs computations akin to a hybrid of an ANN and SVM. This allows it to make use of excess representation units for higher dimensional representation of the input, increasing performance. We see this in practice, as increasing n_R for fixed n_E reduces the loss (bottom-right entries in Fig 5 b).

S1.8. Neuronal dynamics before and after learning

One *apparent* distinction of our model is that there is only one type of error, the one encoded in error unit somata and communicated locally to compartments u^{err} of representation units. Many implementations of PCNs compute two errors in each area: bottom-up and top-down

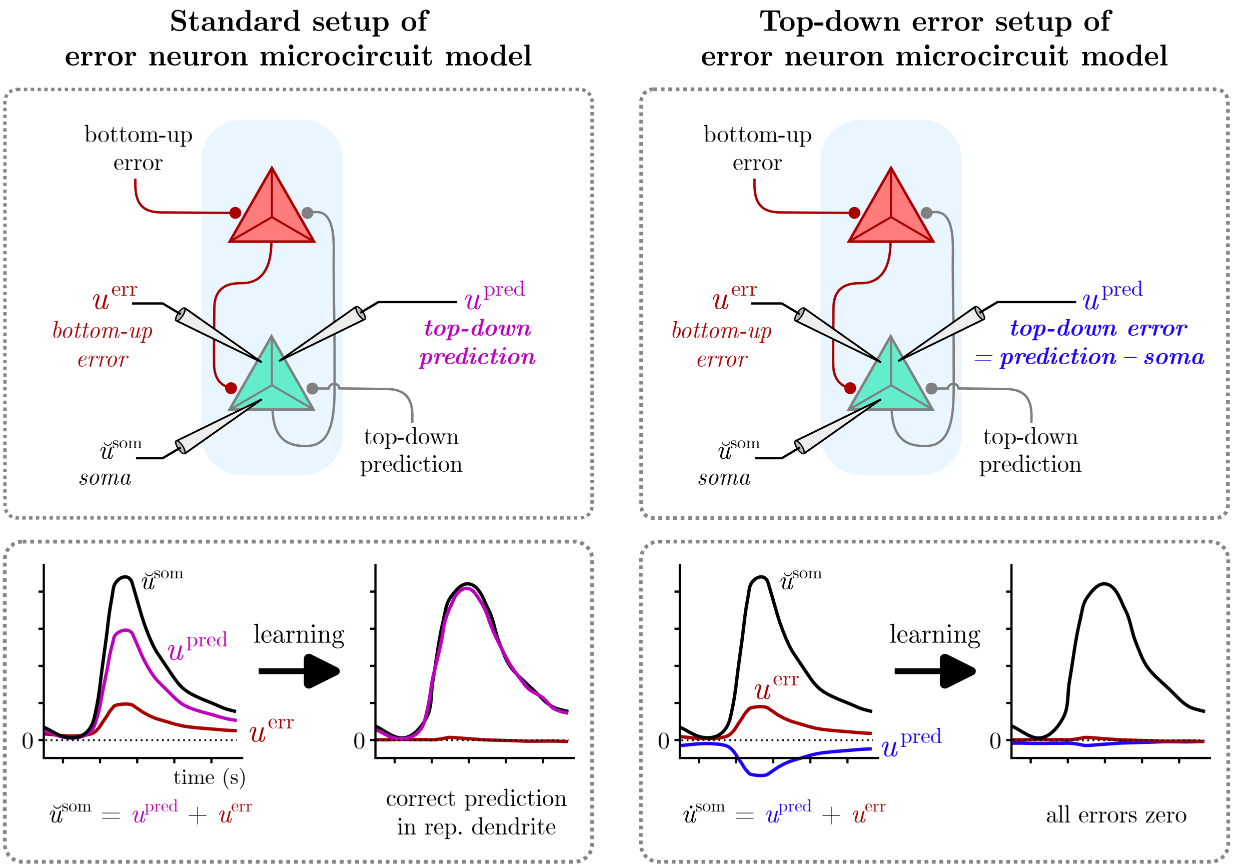

Fig G: Alternative description of our model with top-down and bottom-up errors in representation- and error-receiving dendrites, respectively. Classical predictive coding models, e.g. Rao-Ballard, describe two errors in each area (top-down and bottom-up). In the standard formulation of our model (left), each representation neuron encodes only bottom-up errors (in its error-receiving dendrite); however, the model admits an alternative setup, where predictions are compared to somatic activity on the representation dendrite (right). These two interpretations are computationally fully equivalent, with equal somatic and firing activity as well as learning performance (see S1.3).
The only difference lies in different measurable correlates: in the standard setup, activity in representation dendrites is never zero in the presence of top-down predictions, while the soma integrates top-down predictions with bottom-up errors. In the top-down error setup however, representation dendrites 'explain away' the difference between soma and top-down predictions, leaving only a residual top-down error, which is minimized during learning. We stress that $u^{\text{som}}(t)$ and thus $\breve{u}^{\text{som}}(t)$ remain the same in both interpretations.

errors [25, 29, 69, 125]. For example, in [25] (Box 2), basal errors b encode the usual learning signals (exactly as our u^{err}), while apical errors a are formed by the difference between afferent representations and somatic activity (no direct correlate in our model). This implies different experimental signatures, where basal and apical dendrites both code error signals, which should decrease their activity during learning.

However, note that after convergence of somatic dynamics, both errors are equal up to their sign, $a = -b$, in dendritic hPC (cf. Eq. XI in [25]). I.e., after transient dynamics, only one error signal is in fact encoded, the same in both compartments. Our model allows for an alternative formulation, where top-down errors are also computed on the representation dendrite (see Fig G right). As we demonstrate in S1.3, our model can be rewritten in order to reflect this exactly, and is thus computationally equivalent to the standard formulation, while retaining its scaling advantage over dendritic hPC (cf. Fig 4).

Based on this reformulation, our model (and dendritic hPC) allows for two differing experimental predictions: after transient dynamics have settled, either representation- and error-receiving compartments encode predictions and errors respectively, or they both code the same error signal (with opposing signs, and up to rescaling by conductance couplings), which decreases during learning. Note that the somatic activity is the same in both models in either case (soma = sum of all afferent inputs). An issue of the alternative formulation is the need for the somatic potential to act *subtractively* on the prediction-receiving dendrite (Fig G, right) – it is unclear how biology can implement this, which is why we have chosen to model only one type of error compartment per representation unit in the standard formulation of our dynamics.
